# Amyotrophic Lateral Sclerosis Multiprotein Biomarkers in Peripheral Blood Mononuclear Cells

**DOI:** 10.1371/journal.pone.0025545

**Published:** 2011-10-05

**Authors:** Giovanni Nardo, Silvia Pozzi, Mauro Pignataro, Eliana Lauranzano, Giorgia Spano, Silvia Garbelli, Stefania Mantovani, Kalliopi Marinou, Laura Papetti, Marta Monteforte, Valter Torri, Luca Paris, Gianfranco Bazzoni, Christian Lunetta, Massimo Corbo, Gabriele Mora, Caterina Bendotti, Valentina Bonetto

**Affiliations:** 1 Dulbecco Telethon Institute, Milano, Italy; 2 Department of Molecular Biochemistry and Pharmacology, Mario Negri Institute for Pharmacological Research, Milano, Italy; 3 Department of Neuroscience, Mario Negri Institute for Pharmacological Research, Milano, Italy; 4 Istituto Di Ricovero e Cura a Carattere Scientifico (IRCCS) Fondazione Salvatore Maugeri, Pavia, Italy; 5 National Institute for Occupational Safety and Prevention (ISPESL), Research Center at the IRCCS Fondazione Salvatore Maugeri, Pavia, Italy; 6 IRCCS Fondazione Salvatore Maugeri, Milano, Italy; 7 Department of Oncology, Mario Negri Institute for Pharmacological Research, Milano, Italy; 8 NEuroMuscular Omnicentre (NEMO), Niguarda Ca’ Granda Hospital, Milano, Italy; National Institutes of Health, United States of America

## Abstract

**Background:**

Amyotrophic lateral sclerosis (ALS) is a fatal progressive motor neuron disease, for which there are still no diagnostic/prognostic test and therapy. Specific molecular biomarkers are urgently needed to facilitate clinical studies and speed up the development of effective treatments.

**Methodology/Principal Findings:**

We used a two-dimensional difference in gel electrophoresis approach to identify in easily accessible clinical samples, peripheral blood mononuclear cells (PBMC), a panel of protein biomarkers that are closely associated with ALS. Validations and a longitudinal study were performed by immunoassays on a selected number of proteins. The same proteins were also measured in PBMC and spinal cord of a G93A SOD1 transgenic rat model. We identified combinations of protein biomarkers that can distinguish, with high discriminatory power, ALS patients from healthy controls (98%), and from patients with neurological disorders that may resemble ALS (91%), between two levels of disease severity (90%), and a number of translational biomarkers, that link responses between human and animal model. We demonstrated that TDP-43, cyclophilin A and ERp57 associate with disease progression in a longitudinal study. Moreover, the protein profile changes detected in peripheral blood mononuclear cells of ALS patients are suggestive of possible intracellular pathogenic mechanisms such as endoplasmic reticulum stress, nitrative stress, disturbances in redox regulation and RNA processing.

**Conclusions/Significance:**

Our results indicate that PBMC multiprotein biomarkers could contribute to determine amyotrophic lateral sclerosis diagnosis, differential diagnosis, disease severity and progression, and may help to elucidate pathogenic mechanisms.

## Introduction

Amyotrophic lateral sclerosis (ALS) is an incurable neurodegenerative disorder of unknown cause arising from progressive degeneration of motor neurons and resulting in paralysis and death, usually within 2–4 years from diagnosis. Its incidence is between 1.5 and 2.5 per 100.000 per year: approximately 90% of cases are sporadic and the remaining 10% are familial. The diagnosis is mostly based on clinical assessment with a history of progression of symptoms and is thus made with a delay of about a year from symptom onset, quite likely beyond the therapeutic window of a disease-modifying drug. Moreover, the clinical course varies widely. No ALS biomarkers are currently in clinical use, but they would be valuable to support early diagnosis, monitor disease progression, and assess the efficacy of any new treatment [1].

The pathological process in ALS is now recognized as extending beyond motor neurons [2]–[8], so it can be regarded as a multi-cellular/multi-systemic disease. In particular, peripheral blood mononuclear cells (PBMC) display traits of the disease such as down-regulation of Bcl-2 [9], [10], increased nitrative stress [11], intracellular calcium dysregulation [4] and glutamatergic dysfunction [12], suggesting that they can be a useful source of disease biomarkers.

In a complex disorder it is unlikely that an individual molecule may serve as a clinically useful biomarker. Therefore, proteomic approaches and multiple measurements are likely to be necessary to identify ALS subjects with a worthwhile degree of accuracy. In fact, the most promising candidate biomarkers have been so far combinations of proteins identified in cerebrospinal fluid (CSF) [13]–[15]. However, when the same proteins were searched in plasma either were not present or were not significantly different in comparison with controls [13], [16]. Whereas CSF is considered the ideal source for identifying biomarkers in neurological diseases because of its proximity to the affected tissue, it involves an invasive sampling, that limits large-scale validation studies and thus introduction into clinical practice. PBMC are readily accessible clinical samples and offer a series of advantages over serum/plasma and CSF. Bio-fluids have wide inter-individual variability and a broad range of protein abundance, which make them difficult to analyze by proteomic approaches [17], [18]. The cellular proteome is relatively stable, less complex to analyze and gives direct information on alterations of cellular pathways, hence insights into possible pathogenic mechanisms.

We here reported a proteome-based strategy to identify and validate disease biomarkers in PBMC. By using this procedure we found a panel of proteins that are closely associated with ALS and have high potential in clinical applications and translational medicine. Moreover, our results support the use of PBMC of sporadic ALS (sALS) patients for mechanistic studies.

## Results

### Proteomic analysis and validation

**Figure 1:**
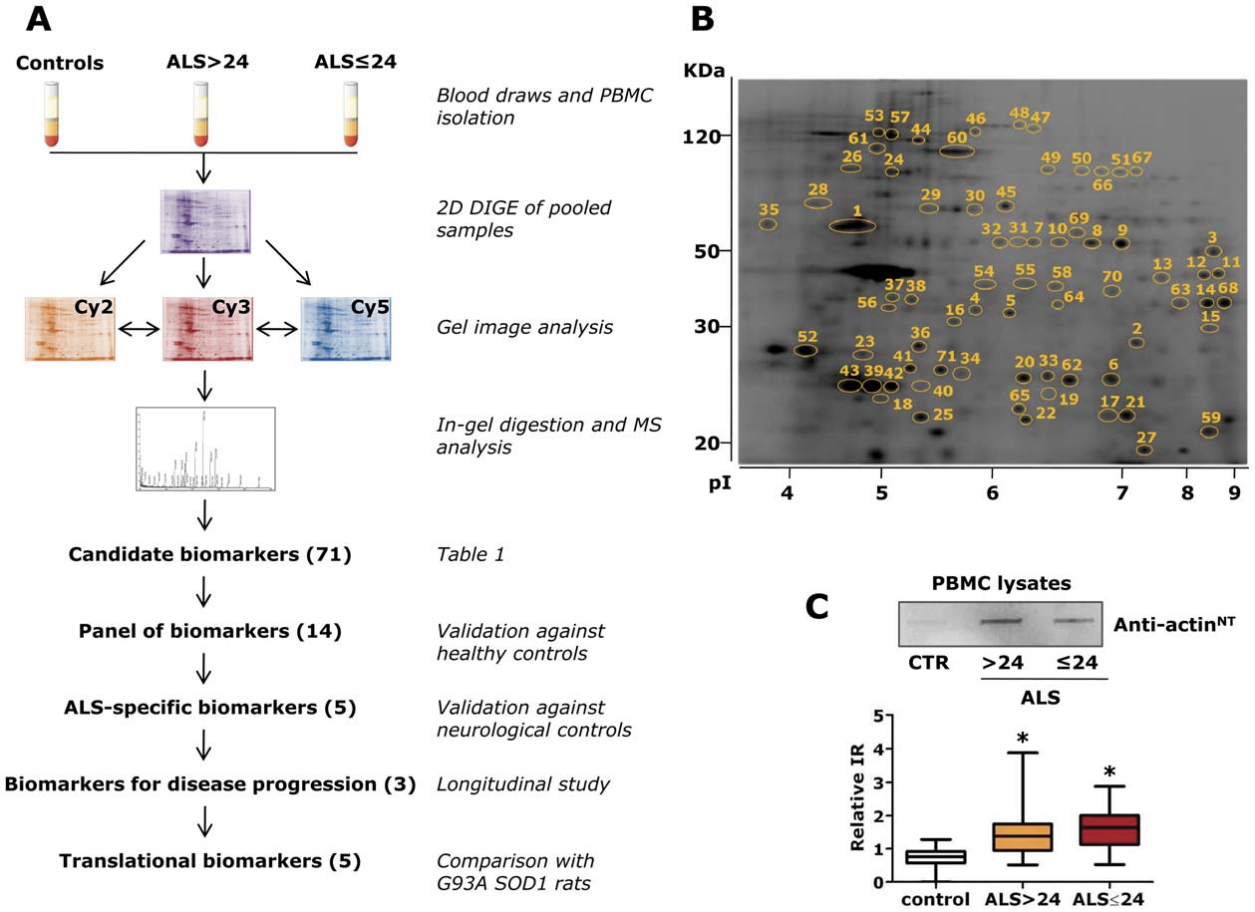
Proteome-based strategy to identify and validate specific biomarkers of ALS in PBMC. (A) PBMC of healthy controls and sALS patients with two levels of disease severity, low, ALS>24, or a high one, ALS≤24, were analyzed by 2D DIGE. Samples were pooled for analysis (n = 11 for each group). The candidate biomarkers, 71 differentially expressed proteins, were identified by mass spectrometry (MS). Validation was done on 14 proteins by dot blot analysis with single samples from an independent set of sALS patients, healthy and non-ALS neurological disorder controls. We obtained five protein biomarkers that significantly distinguish ALS patients from healthy and non ALS neurological controls and three proteins that associate with disease progression. The 14 PBMC protein biomarkers were also measured in G93A SOD1 rats: 5 of them were similarly regulated in the patients and animal model (translational biomarkers). (B) Representative Sypro-Ruby stained 2D gel of the PBMC proteome. The numbered spots correspond to the differentially expressed proteins listed in Table 1; (C) Validation by dot blot analysis was done with commercially available antibodies except for the in-house developed anti-actin^NT^ antibody (Figures S1 and S2). A representative dot blot with the anti-actin^NT^ antibody and quantification of the immunoreactive signals are shown. The box-plot shows relative immunoreactivity (IR) in ALS>24 (n = 30) (yellow box), ALS 24 (n = 30) (red box) patients and healthy controls (n = 30) (white box). The bottom and top of the boxes are the lower and upper quartiles, respectively, the lines inside the boxes indicate the median and the ends of the whiskers represent the minimum and maximum of all the data. *p<0.05 versus controls as assessed by univariate logistic regression.

Figure 1 schematically shows the proteome-based strategy used to identify and validate protein biomarkers of ALS in PBMC. In the first phase, PBMC of healthy controls and sALS patients with two levels of disease severity (Table S1), low, with a ALS functional rating scale revised (ALSFRS-R) score>24 (ALS>24), and high, with a ALSFRS-R score≤24 (ALS≤24), were analyzed by 2D DIGE (Fig. 1A). The analysis, done with 11 pooled samples for each group, detected a total of 129 differential protein spots in the three experimental groups (Table S2). From these spots we used mass spectrometry to identify 71 corresponding proteins (Fig. 1B) that were the candidate protein biomarkers (Tables S3, S4). We classified these proteins in different categories based on their most recognized function (Table 1). Some of the proteins were identified in multiple spots, at lower Mw or different pI than the ones expected (Table S3). These are fragments of the protein or post-translationally modified isoforms.

**Table 1:** The candidate protein biomarkers.

Spot	Protein name	Uniprot^1^
**Energy metabolism**		
1	ATP synthase subunit beta	P06576
2	Triosephosphate isomerase	P60174
3–6	Phosphoglycerate kinase 1	P00558
7–10	Alpha-enolase	Q8WU71
11–13	Fructose-bisphosphate aldolase A	P04075
14	Glyceraldehyde-3-phosphate dehydrogenase	P04406
15	L- Lactate dehydrogenase A chain	P00338
16	L-Lactate dehydrogenase B chain	P07195
**Redox regulation**		
17	Flavin reductase	P30043
18	**Peroxiredoxin-2 (PRDX2)** 2	P32119
19	Peroxiredoxin-6	P30041
20	**Glutathione S-transferase omega-1 (GSTO1)**	P78417
21	Superoxide dismutase [Mn]	P04179
22	Protein DJ-1	Q99497
23	**Chloride intracellular channel protein 1 (CLIC1)**	O00299
**Protein folding and degradation**		
24–25	**Heat shock cognate 71 kDa protein (HSC70)**	P11142
26	78 kDa glucose-regulated protein	P11021
27	**Peptidyl-prolyl cis-trans isomerase A (CypA)**	P62937
28	**Protein disulfide-isomerase (PDI)**	P07237
29–32	**Protein disulfide-isomerase A3 (ERp57)**	P30101
33–34	Endoplasmic reticulum protein ERp29	P30040
35	**Calreticulin (CALR)**	P27797
36	**Proteasome activator complex subunit 1 (PA28a)**	Q06323
**Cytoskeleton-associated**		
37–43	Actin	P60709
44–48	Vinculin	P18206
49–51	Moesin	P26038
52	Tropomyosin alpha-4 chain	P67936
53	Alpha-actinin-1	P12814
54–55	Actin-regulatory protein CAP-G	P40121
56	F-actin capping protein subunit-alpha 1	P52907
57–58	Talin-1	Q9Y490
59	Transgelin-2	P37802
60	Filamin-A	P21333
61	Giantin	Q14789
**Inflammatory response**		
62	Group XIIA secretory phospholipase A2	Q9BZM1
63	Annexin A2	P07355
64	Leukocyte elastase inhibitor	P30740
65	**Interleukin-1 receptor-associated kinase 4 (IRAK4)**	Q9NWZ3
**DNA/RNA binding**		
66–67	**Far upstream element-binding protein 1 (FUBP1)**	Q96AE4
68	**Heterogeneous nuclear ribonucleoproteins A2/B1 (ROA2)**	P22627
69	Probable ATP-dependent RNA helicase DDX41	Q9UJV9
**Others**		
70	AH receptor-interacting protein	O00170
71	Spindle and kinetochore-associated protein 1	Q96BD8

Fold changes and mass spectrometry data are reported in Tables S3, S4.

1 Entry from the Uniprot Knowledgebase database;

2 In bold are the proteins selected for further validations.

**Figure 2:**
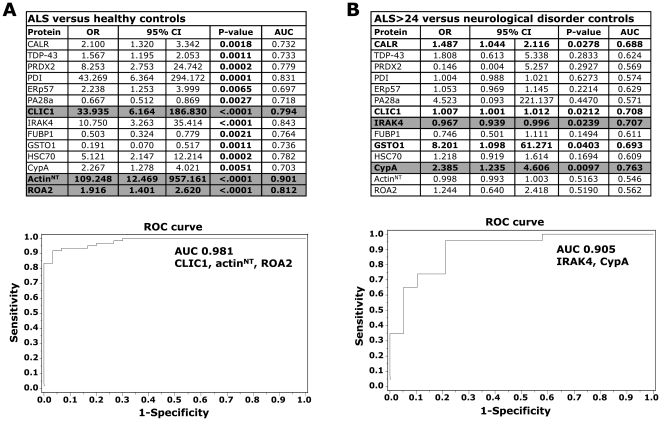
Validation of the 14 selected protein biomarkers versus healthy controls and non-ALS neurological controls: statistical analysis. Validation on single PBMC samples from ALS patients, healthy and non-ALS neurological controls was done by dot blot analysis. Receiver operating characteristics (ROC) curves and analysis of the area under the curve (AUC) were used to find the discriminatory power for proteins showing a significant association with ALS (A,C) or with disease severity (B). Results were expressed as odds ratios (OR) and 95% confidence intervals (95% CI). A 95% CI not including the value of 1 indicates a statistically significant result. All probability values were two-sided and p<0.05 was considered significant. (A) Univariate logistic regression: healthy controls (n = 30) versus ALS, ALS>24 (n = 30) and ALS≤24 (n = 30). All 14 proteins were significantly associated with ALS (*p* values in bold type). CLIC1, actin^NT^ and ROA2 were significant in multivariate analysis (highlighted in grey): a ROC curve for the combination of the three proteins and relative AUC is shown; (B) Univariate logistic regression: ALS>24 (n = 20) versus non-ALS neurological controls (n = 23). CALR, CLIC1, IRAK4, GSTO1, CypA had significant associations with ALS (in bold type). IRAK4 and CypA were significant in multivariate analysis (highlighted in grey): a ROC curve for the combination of the two proteins and relative AUC is shown.

We then validated selected proteins by dot blot analysis on single samples of an independent set of 60 sALS patients, ALS>24 (n = 30) and ALS≤24 (n = 30), and healthy controls (n = 30) (Table S5). From the panel of the candidate biomarkers we validated 12 proteins (PRDX2, GSTO1, CLIC1, HSC70, CypA, PDI, ERp57, CALR, PA28a, IRAK4, FUBP1, ROA2) for the following reasons: they are also expressed in the central nervous system (CNS), they are associated with neurodegenerative processes, some of them specifically with ALS [11], [19]–[24], and are easily detectable by commercially available specific antibodies. In the same validation analysis we also measured actin^NT^, whose level was already found high in PBMC of a small cohort of sALS patients and G93A SOD1 transgenic rats [11] as well as in the spinal cord of the G93A SOD1 mouse model of familial ALS [25]; and TDP-43 identified as the major component of ubiquitinated inclusions in brains of patients with ALS and frontotemporal lobar degeneration [26] and found accumulated in the cytoplasm of PBMC in a group of ALS patients [27]. We confirmed that all 14 proteins can significantly distinguish healthy controls from sALS patients (Fig. 2A, Table 2, Tables S6, S7). As a negative control for the dot blot assay we used SOD1, that was one of the protein spot that did not change in the 2D DIGE analysis. Multivariate logistic analysis showed that CLIC1, actin^NT^ and ROA2 were the proteins most associated with ALS in comparison with healthy controls, with 98% discriminatory power (AUC 0.981) (Fig. 2A). For the combination of these three proteins, the most convenient cut-off generated a sensitivity of 91% and a specificity of 96%. In all 60 sALS patients the levels of the 14 protein biomarkers were independent of sex and age.

**Table 2:** Validation of 14 selected protein biomarkers in patients and healthy controls.

Protein	controls	ALS>24	ALS≤24	ALS>24 vs controls*	ALS≤24 vs controls*	ALS≤24 vs ALS>24*
CALR	2.8±0.7	4.9±2.8	4.4±2.3	1.8	1.6	−1.1
TDP-43	4.1±1.6	5.2±2.1	6.8±2.6	1.3	1.7	1.3
PRDX2	0.9±0.5	1.4±0.7	2.0±1.1	1.6	2.2	1.4
PDI	1.0±0.3	1.6±0.7	1.8±0.7	1.6	1.8	1.1
ERp57	2.5±0.7	3.8±1.4	4.7±2.4	1.5	1.9	1.2
PA28a	4.1±4.5	1.4±1.1	1.6±1.6	−2.9	−2.6	1.1
CLIC1	0.4±0.3	0.8±0.4	0.9±0.5	2.0	2.3	1.1
IRAK4	1.0±0.5	2.2±1.5	2.4±1.5	2.2	2.4	1.1
FUBP1	1.7±1.5	0.6±0.6	0.9±1.0	−2.8	−1.9	1.5
GSTO1	1.2±0.7	0.7±0.5	0.6±0.4	−1.7	−2.0	−1.2
HSC70	0.8±0.5	1.7±1.2	2.3±2.3	2.1	2.9	1.4
CypA	1.4±0.7	3.4±2.7	2.0±1.2	2.4	1.4	−1.7
Actin^NT^	0.7±0.3	1.5±0.8	1.6±0.6	2.1	2.3	1.1
ROA2	2.9±1.8	5.7±2.7	6.4±3.0	2.0	2.2	1.1
SOD1	0.3±0.1	0.3±0.2	0.3±0.1	1.0	1.0	1.0

Validation was done by dot blot assay with the specific antibodies on single PBMC samples from ALS patients, ALS>24 (n = 30) and ALS≤24 (n = 30), and healthy controls (n = 30); mean ± SD of the relative immunoreactivity of the specific antibody normalized to the actual amount of proteins loaded on the membrane as detected after Red Ponceau staining;

*, -fold change as the mean increase (positive) and decrease (negative) in protein concentration in samples from ALS patients in comparison with healthy controls or between the two levels of disease severity.

### CALR, CLIC1, IRAK4, GSTO1 and CypA are ALS-specific biomarkers

We verified the specificity of the 14 biomarkers with PBMC samples from patients with neurological disorders that in some cases may resemble ALS, e.g. some peripheral neuropathies (Table S8). We analyzed our multiprotein biomarkers by dot blot in ALS>24 patients (n = 20) and controls with neurological disorders (n = 23) (Table 3). Univariate logistic analysis showed that CALR, CLIC1, IRAK4, GSTO1 and CypA were associated with ALS (Fig. 2B). Multivariate logistic analysis showed that IRAK4 and CypA were the proteins most associated with ALS rather than neurological disorders, with 91% discriminatory power (AUC 0.905) (Fig. 2B). For the combination of these two proteins, the most convenient cut-off generated a sensitivity of 96% and a specificity of 79%. It is interesting to note that an equally high level of TDP-43 was also detected in patients with neurological disorders (Table 3).

**Table 3:** Validation of the 14 biomarkers in patients and non-ALS neurological controls.

Protein	controls	ALS>24	ALS>24 vs NC*
CALR	4.9±2.8	3.2±1.3	−1.5
TDP-43	2.2±0.6	2.0±0.5	−1.1
PRDX2	0.5±0.2	0.6±0.2	1.2
PDI	0.9±0.4	0.8±0.4	−1.1
ERp57	0.1±0.1	0.1±0.1	1.0
PA28a	0.3±0.2	0.3±0.2	1.0
CLIC1	2.9±2.1	1.5±0.8	−1.9
IRAK4	0.7±0.2	1.0±0.4	1.4
FUBP1	2.0±1.3	2.8±2.0	1.4
GSTO1	0.8±0.6	0.5±0.3	−1.6
HSC70	3.4±2.6	2.3±2.2	−1.5
CypA	4.5±2.2	2.6±1.0	−1.7
Actin^NT^	2.8±1.3	3.0±1.2	1.1
ROA2	1.9±0.9	1.7±1.0	−1.1

Validation was done by dot blot assay with the specific antibodies on single PBMC samples from ALS patients, ALS>24 (n = 20), and non-ALS neurological controls (NC) (n = 23); mean ± SD of the relative immunoreactivity of the specific antibody normalized to the actual amount of proteins loaded on the membrane as detected after Red Ponceau staining;

*, -fold change as the mean increase (positive) and decrease (negative) in protein concentration in samples from ALS patients in comparison with neurological controls.

### CypA, TDP-43 and ERp57 as biomarkers for disease progression

**Figure 3:**
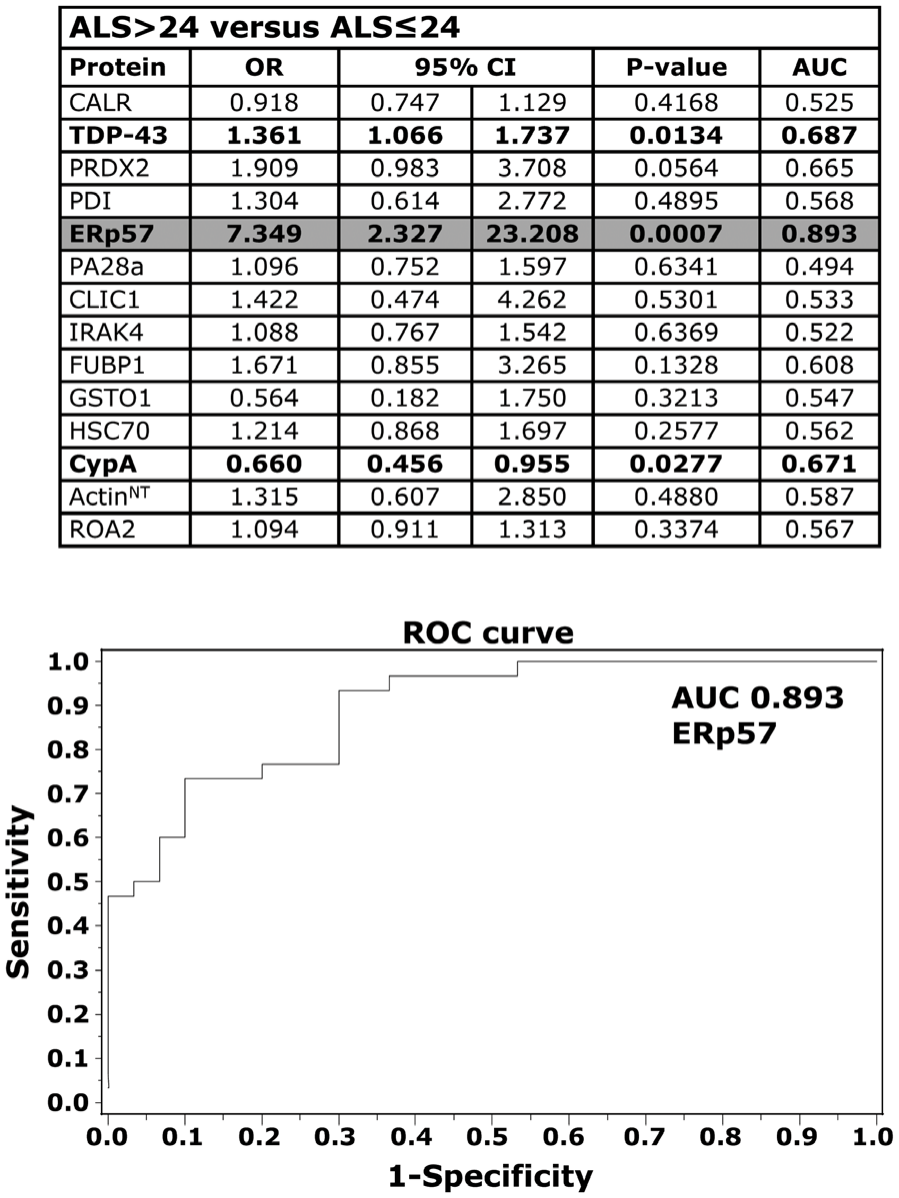
Protein biomarkers that associate with disease severity. Analyses of the 14-protein biomarkers were done on single PBMC samples from ALS patients, ALS>24 (n = 30) and ALS≤24 (n = 30), by dot blot analysis. ROC curves and analysis of the area under the curve (AUC) were used to find the discriminatory power for proteins showing a significant association with disease severity. Results were expressed as odds ratios (OR) and 95% confidence intervals (95% CI). A 95% CI not including the value of 1 indicates a statistically significant result. All probability values were two-sided and p<0.05 was considered significant. Univariate logistic regression: TDP-43, ERp57 and CypA gave a significant association with disease severity (in bold type). Multivariate analysis indicated that ERp57 was the protein most closely associated with disease severity (highlighted in grey). A ROC curve for ERp57 is shown.

**Figure 4:**
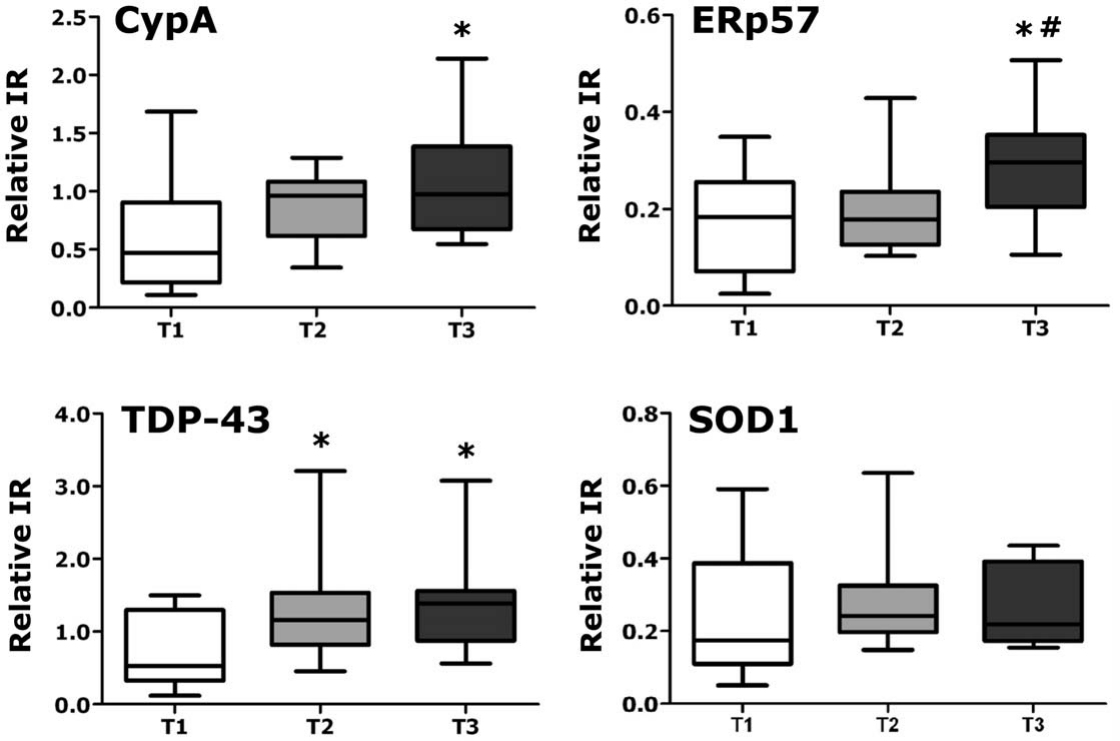
Longitudinal analysis of protein biomarkers CypA, ERp57 and TDP-43. Graphs show relative immunoreactivity (IR) for the indicated proteins measured by dot blot assay in PBMC of sALS patients (n = 13) over a period of six months. PBMC were collected at t = 0 (T1, white column), at t = 3 months (T2, grey column) and at t = 6 months (T3, black column). SOD1 was used as a negative control. Immunoreactivity was normalized to the actual amount of protein loaded, detected after Red Ponceau staining. Data are the means ± SEM of relative immunoreactivity. *, significantly different (*p*<0.05) from T1 as assessed by repeated measures ANOVA followed by Tukey's multiple comparison test; #, significantly different (*p*<0.05) from T2 as assessed by repeated measures ANOVA followed by Tukey's multiple comparison test.

We found that CypA, TDP-43 and ERp57 levels significantly differed in ALS>24 and ALS≤24 patients (Figure 3, Table 2), indicating that these proteins can discriminate between patients with high and low disease severity. Multivariate logistic analysis showed that ERp57 was the most associated with more severe ALS, with 89% discriminatory power (AUC 0.893) (Fig. 3). For ERp57, a threshold of 2.7 relative immunoreactivity generated a sensitivity of 93% and a specificity of 70%. We next examined in a pilot longitudinal study whether CypA, TDP-43 and ERp57 changed over time. We collected three longitudinal PBMC samples from an independent set of 13 sALS patients over a period of six months and used a statistical model for repeated measures to assess the effect of time on protein levels, detected by dot blot assay. The patients had on average a disease duration of 21±10 months, a 33±5 ALSFRS-R score at the first draw and a 28±7 ALSFRS-R score at the third draw (Table S9). The level of all the three protein biomarkers showed a significant increase within six months from the first PBMC collection and for TDP-43 already within 3 months (Fig. 4). As a negative control, in the same set of samples we measured SOD1, which did not significantly change over time (Fig. 4).

### CypA, GSTO1, FUBP1, CLIC1 and actin^NT^ are translational biomarkers of ALS

**Figure 5:**
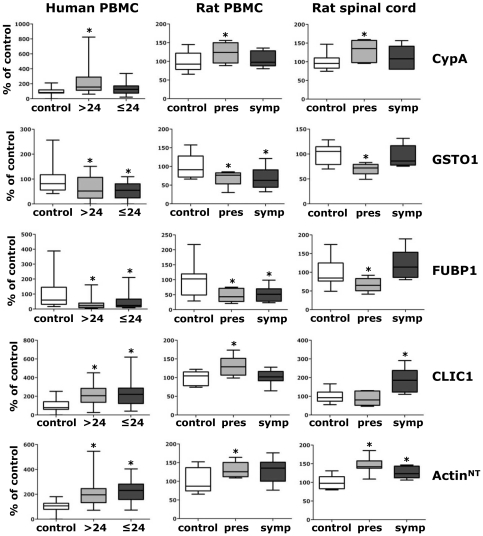
Analysis of protein biomarkers in the G93A SOD1 transgenic rat model: comparison with sALS patients. Human PBMC (left column): box-plot shows relative immunoreactivity for the indicated proteins measured by dot blot assay in ALS>24 (n = 30) (grey box) and ALS≤24 (n = 30) (dark grey box) patients as percentage of healthy controls (control) (n = 30) (white box). *, significantly different (*p*<0.05) from control (univariate logistic regression). Rat PBMC (middle column): box-plot shows relative immunoreactivity for the indicated proteins measured by WB (except for actin^NT^, measured by dot blot) in PBMC lysates (15 µg) of presymptomatic (pres) (n = 6) and symptomatic (symp) G93A SOD1 (n = 6) rats as percentage of non transgenic (control) (n = 8) rats. Protein level values were normalized to actin loading control. *, significantly different (*p*<0.05) from controls (one-way ANOVA followed by Newman-Keuls multiple comparison test). Rat spinal cord (right column): box-plot shows relative immunoreactivity for the indicated proteins measured by dot blot in ventral horn tissue lysates of presymptomatic (pres) (n = 6) and (symp) G93A SOD1 (n = 6) rats as percentage of non transgenic (control) (n = 8) rats. Immunoreactivity was normalized to the actual amount of protein loaded, detected after Red Ponceau staining. *, significantly different (*p*<0.05) from control (one-way ANOVA followed by Newman-Keuls multiple comparison test).

Translational biomarkers are molecules that can be assessed in both human and animal models. To test whether our 14 PBMC proteins were translational biomarkers, their level was measured in PBMC of a transgenic G93A SOD1 rat model of ALS, at pre-symptomatic and symptomatic stages of the disease, in comparison with samples from non transgenic rats by immunoblotting. Five out of fourteen protein biomarkers, CypA, GSTO1, FUBP1, CLIC1 and actin^NT^, showed similar changes in PBMC of sALS patients and transgenic rats (Fig. 5). It is interesting to note that FUBP1 was one of the least specific ALS biomarkers in PBMC of sporadic patients. It is possible that this protein is more associated with SOD1 mutation-induced alterations and is more informative for the mutant SOD1 fALS forms. The translational biomarkers were also investigated in the rat spinal cord at a presymptomatic and symptomatic stages of the disease (Fig. 5). Lumbar spinal cords were sectioned in ventral and dorsal horns to investigate whether proteins alterations were associated or not with motor neuron dysfunction and degeneration. In ventral horns, all of them changed similarly to PBMC already at a presymptomatic stage except for CLIC1, up-regulated only at a symptomatic stage. In dorsal horns, the proteins did not change with the progression of the disease and if compared with controls (Figure S3), indicating that protein alterations observed in ventral horns are specifically associated with motor neuron pathology. In conclusion, CypA, GSTO1, FUBP1, CLIC1 and actin^NT^ are translational biomarkers that might be markers of multi-cellular/multi-systemic alterations underlining pathogenic events both in the human sporadic and animal mutant SOD1-linked disease forms.

## Discussion

A number of gene expression studies have demonstrated the utility of PBMC as a source of biomarkers in neurological disorders [28]–[30]. This is the first study that describes a highly feasible strategy to identify and validate multiprotein biomarkers in PBMC, potentially applicable to several neurological/neurodegenerative diseases. Protein profiles are directly connected to changes in molecular pathways related to health and disease, therefore have the potential to accurately monitor the progression of the disease or the response to a treatment.

We have previously shown that PBMC undergo immunophenotypic changes in sALS patients [10]. We have now identified a panel of protein biomarkers in these cells that are associated with sALS with high discriminatory power. These PBMC proteins are easily measurable in large-scale immunoassays aimed at developing diagnostic/prognostic tests for clinical use. The great advantages of such an *in vitro* test is low invasiveness for the patient compared to CSF tests, the consequent greater availability of samples for large clinical studies, including longitudinal ones, and the simple laboratory procedures involved.

The ideal diagnostic marker should detect disease before clinical diagnosis, which is highly challenging for a rare and sporadic disease. ALS patients very often see the specialized neurologist only months after the first symptoms, when they are unquestionably ill. It is therefore very difficult to test and validate the applicability of biomarkers in preclinical diagnosis. Our PBMC protein biomarkers seems to be promising to support prompt clinical diagnosis, since all the 14 validated proteins can distinguish with high significance ALS patients with low disability from healthy controls (Table S6). PBMC biomarkers could be measured on patients after as early as 5 months up to 108 months from symptom onset, and with high (from 45) and low (up to 10) ALSFRS-R scores (Table S5, S8, S9). Now large-scale validations are needed, in which data will be verified in a prospective cohort with analysis of the biomarkers at the time of symptom presentation. In the G93A SOD1 rat model of ALS, alterations of the level of CypA, GSTO1, FUBP1, CLIC1 and actin^NT^ are detected before disease onset. It is possible that some of these biomarkers may predict the onset of SOD1-linked familial ALS. This potential application will be also considered in future analysis.

Up to 10% of patients initially diagnosed as having ALS are false positive [31], [32]. A similar percentage is false negative and undergoes inappropriate medical or surgical procedures [33], [34]. Thus, there is a need for biomarkers that distinguish ALS with high accuracy from neurological disorders that in some cases may resemble it, e.g. some peripheral neuropathies that are treatable and do not have a fatal prognosis. We found that there are PBMC protein biomarkers that significantly distinguish ALS from the group of neurological disorders considered in our study. These are chaperones (CALR, CypA), proteins involved in redox homeostasis (GSTO1, CLIC1) and immune responses (IRAK4). IRAK4, which has no previous association with ALS, has a central function in innate immunity [35].

Finally, multivariate analysis helped us in defining the most convenient combination of protein biomarkers that could be potentially used in clinics (i) to support diagnosis (CLIC1, actin^NT^ and ROA2), (ii) to contribute to differential diagnosis of ALS from other neurological conditions (CypA and IRAK4), and (iii) to determine disease severity (ERp57).

There are no precise measures of ALS disease progression that allow for short-term monitoring of the disease and assessment of treatment efficacy. In clinical trials survival time is therefore used as the primary measure of outcome. This requires large number of patients followed over a long period of time making ALS clinical trials very expensive. A panel of biomarkers that can reliably assess disease progression would enable a substantial reduction of the costs of the clinical trial and accelerate therapy development in ALS. ERp57, CypA and TDP-43, that were able to discriminate between patients with high and low disease severity, were selected for a pilot longitudinal study and proved to be good candidates for such applications. Large longitudinal studies are now needed to further validate the use of these proteins in clinical practice.

The pathogenesis of sALS is largely unknown. The concept of PBMC as a window into the CNS has been already proposed for several neurological disease states [28]–[30], [36], [37]. CNS and immune cells communicate through multiple mechanisms and have several similarities in receptor expression and transduction processes [38]. We therefore hypothesized that PBMC protein profiles could help to elucidate pathways underlying ALS etiology. Indeed, some of the protein biomarkers identified in PBMC of sALS patients were previously found as hallmarks of disease in CNS. Studies in spinal cord tissues of sALS patients showed that PDI and ERp57 were up-regulated [20], CypA and HSC70 were accumulated in the detergent-insoluble fraction [39], HSC70 was present in hyaline inclusions [24], and TDP-43 was identified as the major component of the ubiquitinated inclusions [26].

The protein profile changes detected in PBMC of ALS patients are suggestive of possible pathogenic mechanisms. For instance, up-regulation of endoplasmic reticulum (ER) chaperones (PDI, ERp57, CALR) is a typical cellular response to ER stress that triggers the unfolded protein response leading eventually to cell death [40]. The increased level of a nitrotyrosine-linked protein, actin^NT^, is indicative of nitrative stress [41]. Alterations in CypA, GSTO1, PRDX2 suggest disturbances in cellular redox regulation [42]–[45]. It is important to note that all these pathogenic alterations were previously reported in the spinal cord of sALS patients and mutant SOD1 animal models [19], [20], [25], [46]–[49]. ROA2 belongs to the family of heterogeneous nuclear ribonucleoproteins that participates to several RNA-related biological processes such as transcription, pre-mRNA processing, mRNA transport to the cytoplasm and translation [50]. It is also a binding partner of TDP-43 and seems to be crucial for at least one of its putative functions [51]. The up-regulation of ROA2 and TDP-43 in PBMC of both ALS>24 and ALS≤24 patients may underline aberrant RNA processing events that are now emerging as central in ALS and other neurological disorders [52]. These specific intracellular alterations would not be detectable from the protein profiles of CSF or plasma. Neuroinflammation, which is characterized by activated microglia and infiltrating peripheral blood immune cells, is a prominent pathological feature in ALS [53]. It is possible to speculate that such consistent protein profile changes in immune cells may influence their infiltration and/or interaction with microglia and neurons, thus upsetting the delicate balance between neuroprotection and neurotoxicity. In summary, all these data endorse the use of PBMC for further *in vitro* mechanistic studies. Experimental models for the sporadic form are not available and all mechanistic studies are done with transgenic cells and animals expressing one of the mutant genes linked to familial ALS. Studies with PBMC would have the advantage to consider the influence of the genetic background of the patient.

Translational biomarkers, that link responses between human and animal model, are of particular interest because their role in the pathogenesis can be investigated in detail in the animal model where they can also offer important preliminary information for clinical trials. We found that CypA, GSTO1, FUBP1, CLIC1 and actin^NT^ are translational biomarkers. Moreover, all of them except for CLIC1 are altered in the ventral horn spinal cord of SOD1 G93A rats before disease onset, suggesting a possible involvement in pathways that trigger the disease. Further mechanistic studies in the animal models with these proteins are now warranted.

In conclusion, we identified and verified a panel of highly promising protein biomarkers of ALS in PBMC that may be useful in clinical studies, helping elucidate pathogenic mechanisms and pin-pointing pathways to tackle for future therapeutic interventions. The fact that our protein biomarkers are easily measurable in accessible clinical samples using straightforward immunoassays makes them attractive candidates for true multi-centric large-scale validations and eventually introduction into clinical practice.

## Materials and Methods

### Subjects

The study was approved by the Ethical Committees of all the Centers involved in the study, IRCCS Fondazione S. Maugeri, in Milano and Pavia, NEMO-Niguarda Ca’ Granda Hospital, Milano, and Transfusion Medical Centre at the IRCCS Policlinico S. Matteo, Pavia, and written informed consent was obtained from all participating subjects. In this study we included 94 sALS patients and 64 controls (41 healthy, 23 with non-ALS neurological disorders). Patients with definite ALS, according to revised El Escorial criteria, were examined and blood samples were collected at the IRCCS Fondazione S. Maugeri, in Milano and Pavia, Italy. To evaluate the level of disability of the patients the ALSFRS-R score was used [54]. The patients were arbitrary divided into two groups according to the ALSFRS-R score, >24 or ≤24. None of the sALS patients had systemic inflammatory conditions as detected by the erythrocyte sedimentation rate and total blood cell count. Sixty-five percent of the patients were treated with riluzole. For the longitudinal study PBMC samples were collected every three months over a period of six months at the NEMO, Niguarda Ca’ Granda Hospital. Blood samples of non-ALS neurological disorder controls were provided by the NEMO and the IRCCS Fondazione S. Maugeri, in Milano and Pavia. None of the patients was receiving drugs that might interfere with total blood cell count. The characteristics of ALS patients and non-ALS neurological disorder controls are further described in Tables S1, S5, S8 and S9. Blood samples of healthy donors were provided by the Transfusion Medical Centre at the IRCCS Policlinico S. Matteo, Pavia.

### PBMC

Samples of peripheral venous blood from patients and controls were collected in EDTA pre-coated vials (Vacuette K3E K3EDTA, Greiner bio-one). PBMC were isolated from EDTA-blood by Ficoll-Hypaque (Ficoll-Plaque™ Plus, GE Healthcare) density gradient centrifugation. Mononuclear cells were harvested from the interface and washed three times with RPMI 1640 medium (EuroClone). Platelets were eliminated by an additional wash and centrifugation at 200× g for 10 min. Patients' and controls' PBMC were stored as pellets at −80°C. Just before analysis PBMC proteins were obtained by cell lysis in 20 mM Tris-HCl pH 7.5, 0.1% NP40 and 0.1% SDS supplemented with Protease Inhibitors (Sigma). Proteins were quantified by the BCA protein assay (Pierce).

### Two-dimensional difference in gel electrophoresis (2D DIGE)

PBMC proteins were prepared for 2D DIGE analysis. Three pools of 25 µg from 11 healthy controls, 11 ALS>24 and 11 ALS≤24 patients were methanol-precipitated. Proteins were then dissolved in 30 mM Tris-HCl pH 8.5, 7 M urea, 2 M thiourea, CHAPS 4% (w/v) and Cydye-labeled according to the manufacturer's instructions (GE Healthcare) with minor modifications. Briefly, 25 µg of each pool was labeled with 200 pmol of Cy3 or Cy5 dye for 30 min in ice in the dark. To exclude preferential labeling of the dyes, each sample was also reverse labeled. As an internal standard, aliquots of each pool were mixed and labeled with Cy2 dye. Labeled samples were then resuspended in Destreak Solution™ (GE Healthcare) with IPG buffer pH 3–10 NL 0.5% v/v (GE Healthcare) added and loaded into 7 cm-IPG strips pI range 3–10NL (GE Healthcare). Isoelectrofocusing was done on an IPGphor apparatus (GE Healthcare) with the following protocol: 30 V for 270 Vhrs, 200 V for 200 Vhrs, 2000 V for 2000 Vhrs, a linear gradient of 3500 V for 1375 Vhrs, 3500 V for 7000 Vhrs, a linear gradient of 8000 V for 8625 Vhr, 8000 V for 32000 Vhr and forever at 30 V. SDS-PAGE was done using precast 10% polyacrylamide SDS gel (Invitrogen). Four 2D gels were run with the three experimental groups: healthy controls and ALS patients, ALS>24, and ALS≤24. Each gel contained two experimental groups, one Cy3-labelled, the other Cy5-labelled plus the Cy2-labelled internal standard. Gel images were captured by the laser scanner Molecular Imager FX (Bio-Rad). Image analysis was done with Progenesis PG240 v2006 software (Nonlinear Dynamics). For each spot the normalized volume was standardized against the intra-gel standard, dividing the value for each spot normal volume by the corresponding internal standard spot normal volume within each gel. The values for each spot in each group were expressed as the mean of the Cy3- and Cy5-labelled analyses. The values for ALS patients were reported as fold change: higher (positive) or lower (negative) spot volume of the samples from ALS patients in comparison with healthy controls (Table S4).

### Protein identification

Protein spots were located and excised from 2D gels with the EXQuest™ spot cutter (Bio-Rad). Spots were processed and gel-digested with modified trypsin from bovine pancreas (Roche) and identified by mass spectrometry (MS), essentially as previously described [25]. Digestion, desalting and concentration with ZipTip® pipette tips with C18 resin (Millipore) and MALDI target deposition were carried out on an automated protein digestor DigestPro MS (Intavis AG). Peptide mass fingerprinting and tandem mass spectrometry (MS/MS) were done on a 4800 MALDI TOF/TOF mass spectrometer (Applied Biosystems). The mass spectra were internally calibrated with trypsin autolysis fragments. The five most abundant precursor ions, out of the exclusion mass list (ions from human keratin and trypsin), were selected for MS/MS analysis. The combined MS and MS/MS data were submitted by GPS Explorer v.3.6 software (Applied Biosystems) to the MASCOT database search engine (Version 2.1, Matrix Science) and searched with the following parameters: Uniprot_Swissprot 57.8 database over all *Homo sapiens* protein sequences deposited, no fixed modifications, as possible modifications carboamidomethylation of cysteine and oxidation of methionine, 1 missed trypsin cleavage, a mass tolerance of ±0.1 Da for the peptide masses and ±0.3 Da for the MS/MS fragment ion masses. A protein was regarded as identified if the MASCOT protein score, based on the combined MS and MS/MS data, was above the 5% significance threshold for the database [55]. Some of the proteins were identified by liquid chromatography-(LC)-MS/MS using the microfluid chip-based technology for nanoelectrospray coupled to an ion trap mass spectrometer (Agilent 1200 LC/MSD Trap XCT) as previously described [56]. Data were acquired sequentially in MS mode (scan range 200–2000 amu) and in data-dependent mode, automatically recording the MS/MS spectra of the four most abundant ions in every scan cycle. Data files of all MS/MS spectra in a LC run were merged, and submitted as an “mgf” file (BioWorks Rev 3.1 SR1, Thermo Scientific) to the MASCOT database search engine in MS/MS Ion Search mode. A protein was regarded as identified if MASCOT individual ion scores were >36, which indicate identity or extensive homology (p<0.05), for at least three matched peptides. Search parameters were the same as above.

### Anti-nitrated actin (actin^NT^) antibody preparation

Purified human non-muscle actin (>99% pure; Cytoskeleton, Inc.) (1.5 mg/mL) was incubated for 24 hours at room temperature in a nitration solution containing 20 mM sodium acetate pH 5.6, 9 µM FeCl_3_, 10 mM NaNO_2_, 0.3% H_2_O_2_. Nitration of the protein was verified by Western blotting (WB) with the anti-nitrotyrosine antibody (clone HM.11; Hycult Biotechnology). Actin^NT^ was used as antigen in rabbits for raising polyclonal antibodies at Eurogentec S.A. LIEGE Science Park (Belgium). Anti-actin^NT^ antibody was characterized as described in Figures S1 and S2.

### Dot blot analysis

Aliquots (3 µg) of PBMC samples were loaded on nitrocellulose membrane, Trans-Blot Transfer Medium (Bio-Rad), by vacuum deposition on the Bio-Dot SF blotting apparatus (Bio-Rad). Membranes were probed overnight with primary antibodies: rabbit polyclonal anti-CALR (1∶5000), rabbit polyclonal anti-PDI (1∶4000) and mouse monoclonal anti-ERp57 (1∶500) from StressGen, mouse monoclonal anti-HSC70 (1∶1000), goat polyclonal anti-PRDX2 (1∶2000), goat polyclonal anti-PA28a (1∶2000), mouse monoclonal anti-CLIC1 (1∶2500), goat polyclonal anti-IRAK4 (1∶1000) and rabbit polyclonal anti-FUBP1 (1∶1000) from Santa Cruz Biotechnology, mouse monoclonal anti-GSTO1 (1∶500) and mouse monoclonal anti-ROA2 (1∶2500) from Abnova, rabbit polyclonal anti-CypA (1∶2500) and rabbit polyclonal anti-hSOD1 (1∶2000) from Upstate Biotechnology, rabbit polyclonal anti-actin^NT^ (1∶7500) developed in-house, and rabbit polyclonal anti-TDP-43 (1∶2000), kindly provided by Francisco Baralle, ICGEB, Trieste, Italy, and then with secondary anti-mouse, anti-rabbit or anti-goat peroxidase-conjugated secondary antibodies (Santa Cruz Biotechnology). Blots were developed by Immobilon Western Chemiluminescent HRP Substrate (Millipore) on the ChemiDoc XRS system (Bio-Rad). Densitometry was done with Progenesis PG240 v2006 software (Nonlinear Dynamics). Immunoreactivity was normalized to the actual amount of proteins loaded on the membrane as detected after Red Ponceau staining (Fluka).

### Statistical analysis

Continuous variables such as age and all data of the considered proteins were described using ‘standard’ statistics (mean, standard deviation), and relative and absolute frequencies were used for categorical variables. Spearman correlation analysis was done to assess the associations between the 14 proteins. Univariate and multivariate logistic regression models were also built to identify the level of association between proteins and ALS patients. Receiver operating characteristics (ROC) curves and analysis of the area under the curve (AUC) were used to find the discriminatory power for proteins showing a significant association with ALS or disease severity. Results were expressed as odds ratios (OR) and 95% confidence intervals (95% CI). A 95% CI not including the value of 1 indicates a significant result. Dependence on sex and age (at the time of PBMC collection) was analyzed by two-way ANOVA. All probability values were two-sided and p<0.05 was considered statistically significant. Version 9.1 of the SAS statistical software (SAS Institute, Inc, Cary, NC, USA) and version 5.03 of the GraphPad Prism software (GraphPad Software, Inc, La Jolla, CA, USA) were used.

### Analysis of protein biomarkers in the rat model

Procedures involving animals and their care were conducted in conformity with the institutional guidelines that are in compliance with national (D.L. No. 116, Suppl. 40, Feb. 18, 1992 Circolare No. 8, G.U., 14 luglio 1994) and International laws and policies (EEC Council Directive 86/609. OJ L 358,1, Dec. 12, 1987; *NIH Guide for Care and use of Laboratory Animals, U.S. National Research Council, 1996*). They were reviewed and approved by the Mario Negri Institute Animal Care and Use Committee that includes *ad hoc* members for ethical issues (ID 33/01-D Appl3). Non transgenic and transgenic rats expressing a high copy number of mutant human SOD1 with Gly-93-Ala substitution were bred and maintained at the Mario Negri Institute for Pharmacological Research, Milan, Italy. Animals were housed at 21±1°C with relative humidity 55±10% and 12 h of light. Food (standard pellets) and water were supplied *ad libitum*. Transgenic rats were identified with PCR on DNA from tail biopsies. G93A SOD1 rats were sacrificed at presymptomatic (15 weeks of age) and early-symptomatic (20 weeks of age) stages of disease. Non transgenic rats were used as controls (20 weeks of age). Blood was sampled directly by intracardiac puncture from rats and collected in EDTA pre-coated vials (Vacuette K3E K3EDTA, Greiner bio-one). PBMC were isolated from blood, washed and stored as pellets at −80°C in the same way as the human samples. PBMC proteins were obtained by cell lysis in 50 mM Tris-HCl, pH 7.5, 2% (w/v) CHAPS, 37.5 U benzonase (Merck), supplemented with Protease Inhibitors (Sigma). Proteins were quantified by the BCA protein assay (Pierce). Proteins were separated by SDS-PAGE on precast 12% Criterion XT Bis-Tris gels (Bio-Rad) and immunoblotted, as described [39]. The blots were probed with primary and secondary antibodies, as described for dot blot assay of the human samples. Blots were also probed with an antibody that recognizes actin (mouse monoclonal, 1∶1000 dilution, Chemicon) for loading control and developed by Immobilon Western Chemiluminescent HRP Substrate (Millipore) on the ChemiDoc XRS system (Bio-Rad). Densitometry was done with Progenesis PG240 v2006 software (Nonlinear Dynamics). Spinal cords were removed and the lumbar tract was cut into ventral and dorsal horn sections on a cryostat. Ventral and dorsal horn sections were suspended in 1% SDS (5 µL/mg), sonicated and boiled for 10 min. Homogenates were centrifuged at 12000 RPM and supernatants analyzed by dot blot, as described for the human samples except for the FUBP1 and PA28a analyses where the mouse monoclonal anti-FUBP1 antibody (1∶500) from Santa Cruz Biotechnology and the rabbit polyclonal anti-PA28a antibody (1∶1000) from Cell Signaling were used.

## Supporting Information

Figure S1 **Anti-nitrated actin (actin^NT^) antibody preparation and characterization by dot blot assay.** Purified human actin was nitrated *in vitro* and used as antigen in rabbits for raising polyclonal antibodies. The rabbit polyclonal antiserum was tested by dot blot with actin, actin^NT^, bovine serum albumin (BSA) and BSA^NT^, prepared by the same procedure as actin^NT^. Figure S1 shows that anti-actin^NT^ does not efficiently recognize another nitrated protein and has more than three times affinity for actin^NT^ of unmodified actin. 3 µg of actin, actin^NT^, BSA and BSA^NT^ were loaded in each slot on the nitrocellulose membrane. The membrane was probed overnight with the polyclonal antiserum diluted 1∶7500. Immunoreactivity was normalized to the actual amount of protein loaded on the membrane as detected after Red Ponceau staining.
(TIF)

Figure S2 **Characterization of the anti-actin^NT^ antibody.** The anti-actin^NT^ antibody was further characterized to determine the specificity of the immunoreactivity against PBMC lysates from controls and ALS patients by Western blotting. Figure S2 shows that the antiserum recognized a band at 42 kDa, which is the expected Mw for actin^NT^, but also other bands at higher Mw. These are probably polymerized actin forms. These high-Mw species are essentially only detected in the patients and make this antibody especially useful to distinguish ALS patients from controls, also in a dot blot assay, as shown in Figure 1. Equal amounts of PBMC lysates (30 µg) from healthy controls and ALS patients were analyzed. A representative experiment is shown. The PVDF membrane was probed first with the anti-actin antibody (mouse monoclonal, 1∶1000 dilution, Chemicon), and the signal was revealed with a goat anti-mouse Qdot® 800-conjugated secondary antibody (Invitrogen, 1∶1000 dilution) on a laser scanner Molecular Imager FX (Bio-Rad), then with the anti-actin^NT^ antibody, and the signal was revealed with an anti-rabbit peroxidase-conjugated secondary antibody/chemiluminescent HRP Substrate (Millipore) on a ChemiDoc XRS system (Bio-Rad).
(TIF)

Figure S3 **Analysis of protein biomarkers in dorsal horn spinal cord of the G93A SOD1 transgenic rat.** Bars show relative immunoreactivity for the indicated proteins measured by dot blot in ventral horn tissue lysates of presymptomatic (pres, grey bars) (n = 6) and (symp, dark grey bars) G93A SOD1 (n = 6) rats and non transgenic (control, white bars) (n = 8) rats. Immunoreactivity was normalized to the actual amount of protein loaded, detected after Red Ponceau staining. None of the protein levels were significantly different from control (one-way ANOVA followed by Newman-Keuls multiple comparison test).
(TIF)

Table S1 **Main characteristics of healthy individuals and sALS patients used in the proteomic 2D DIGE analysis.**
(DOC)

Table S2 **Spot volume changes in 2D DIGE analysis.**
(DOC)

Table S3 **Candidate protein biomarkers identified by mass spectrometry.**
(DOC)

Table S4 **Candidate protein biomarkers: 2D DIGE quantitative analysis.**
(DOC)

Table S5 **Main characteristics of healthy individuals and sALS patients used for the validation analysis (** **Figure 2A** **and** **Figure 3** **).**
(DOC)

Table S6 **Univariate logistic regression: controls versus ALS>24.**
(DOC)

Table S7 **Univariate logistic regression: controls versus ALS≤24.**
(DOC)

Table S8 **Characteristics of sALS patients and non-ALS neurological controls used for the validation analysis (** **Figure 2B** **).**
(DOC)

Table S9 **Characteristics of sALS patients used for the longitudinal study (** **Figure 4** **).**
(DOC)

## References

[1] Turner MR, Kiernan MC, Leigh PN, Talbot K (2009). Biomarkers in amyotrophic lateral sclerosis.. Lancet Neurol.

[2] Aguirre T, Van Den Bosch L, Goetschalckx K, Tilkin P, Mathijs G (1998). Increased sensitivity of fibroblasts from amyotrophic lateral sclerosis patients to oxidative stress.. Ann Neurol.

[3] Clement AM, Nguyen MD, Roberts EA, Garcia ML, Boillee S (2003). Wild-type nonneuronal cells extend survival of SOD1 mutant motor neurons in ALS mice.. Science.

[4] Curti D, Malaspina A, Facchetti G, Camana C, Mazzini L (1996). Amyotrophic lateral sclerosis: oxidative energy metabolism and calcium homeostasis in peripheral blood lymphocytes.. Neurology.

[5] Dobrowolny G, Aucello M, Rizzuto E, Beccafico S, Mammucari C (2008). Skeletal muscle is a primary target of SOD1G93A-mediated toxicity.. Cell Metab.

[6] Ono S (2000). The skin in amyotrophic lateral sclerosis.. Amyotroph Lateral Scler Other Motor Neuron Disord.

[7] Wong M, Martin LJ (2010). Skeletal Muscle-Restricted Expression of Human SOD1 Causes Motor Neuron Degeneration in Transgenic Mice.. Hum Mol Genet.

[8] Beers DR, Henkel JS, Xiao Q, Zhao W, Wang J (2006). Wild-type microglia extend survival in PU.1 knockout mice with familial amyotrophic lateral sclerosis.. Proc Natl Acad Sci U S A.

[9] Cova E, Cereda C, Galli A, Curti D, Finotti C (2006). Modified expression of Bcl-2 and SOD1 proteins in lymphocytes from sporadic ALS patients.. Neurosci Lett.

[10] Mantovani S, Garbelli S, Pasini A, Alimonti D, Perotti C (2009). Immune system alterations in sporadic amyotrophic lateral sclerosis patients suggest an ongoing neuroinflammatory process.. J Neuroimmunol.

[11] Nardo G, Pozzi S, Mantovani S, Garbelli S, Marinou K (2009). Nitroproteomics of peripheral blood mononuclear cells from patients and a rat model of ALS.. Antioxid Redox Signal.

[12] Poulopoulou C, Davaki P, Koliaraki V, Kolovou D, Markakis I (2005). Reduced expression of metabotropic glutamate receptor 2mRNA in T cells of ALS patients.. Ann Neurol.

[13] Ganesalingam J, An J, Shaw CE, Shaw G, Lacomis D (2010). Combination of neurofilament heavy chain and complement C3 as CSF biomarkers for ALS.. J Neurochem.

[14] Mitchell RM, Freeman WM, Randazzo WT, Stephens HE, Beard JL (2009). A CSF biomarker panel for identification of patients with amyotrophic lateral sclerosis.. Neurology.

[15] Pasinetti GM, Ungar LH, Lange DJ, Yemul S, Deng H (2006). Identification of potential CSF biomarkers in ALS.. Neurology.

[16] Mitchell RM, Simmons Z, Beard JL, Stephens HE, Connor JR (2010). Plasma biomarkers associated with ALS and their relationship to iron homeostasis.. Muscle Nerve.

[17] Hu Y, Malone JP, Fagan AM, Townsend RR, Holtzman DM (2005). Comparative proteomic analysis of intra- and interindividual variation in human cerebrospinal fluid.. Mol Cell Proteomics.

[18] Zhang J (2007). Proteomics of human cerebrospinal fluid - the good, the bad, and the ugly.. Proteomics Clin Appl.

[19] Atkin JD, Farg MA, Turner BJ, Tomas D, Lysaght JA (2006). Induction of the unfolded protein response in familial amyotrophic lateral sclerosis and association of protein-disulfide isomerase with superoxide dismutase 1.. J Biol Chem.

[20] Atkin JD, Farg MA, Walker AK, McLean C, Tomas D (2008). Endoplasmic reticulum stress and induction of the unfolded protein response in human sporadic amyotrophic lateral sclerosis.. Neurobiol Dis.

[21] Kato S, Kato M, Abe Y, Matsumura T, Nishino T (2005). Redox system expression in the motor neurons in amyotrophic lateral sclerosis (ALS): immunohistochemical studies on sporadic ALS, superoxide dismutase 1 (SOD1)-mutated familial ALS, and SOD1-mutated ALS animal models.. Acta Neuropathol.

[22] Massignan T, Casoni F, Basso M, Stefanazzi P, Biasini E (2007). Proteomic analysis of spinal cord of presymptomatic amyotrophic lateral sclerosis G93A SOD1 mouse.. Biochem Biophys Res Commun.

[23] van de Giessen E, Fogh I, Gopinath S, Smith B, Hu X (2008). Association study on glutathione S-transferase omega 1 and 2 and familial ALS.. Amyotroph Lateral Scler.

[24] Watanabe M, Dykes-Hoberg M, Culotta VC, Price DL, Wong PC (2001). Histological evidence of protein aggregation in mutant SOD1 transgenic mice and in amyotrophic lateral sclerosis neural tissues.. Neurobiol Dis.

[25] Casoni F, Basso M, Massignan T, Gianazza E, Cheroni C (2005). Protein nitration in a mouse model of familial amyotrophic lateral sclerosis: possible multifunctional role in the pathogenesis.. J Biol Chem.

[26] Neumann M, Sampathu DM, Kwong LK, Truax AC, Micsenyi MC (2006). Ubiquitinated TDP-43 in frontotemporal lobar degeneration and amyotrophic lateral sclerosis.. Science.

[27] De Marco G, Lupino E, Calvo A, Moglia C, Buccinna B (2011). Cytoplasmic accumulation of TDP-43 in circulating lymphomonocytes of ALS patients with and without TARDBP mutations.. Acta Neuropathol.

[28] Achiron A, Gurevich M (2006). Peripheral blood gene expression signature mirrors central nervous system disease: the model of multiple sclerosis.. Autoimmun Rev.

[29] Borovecki F, Lovrecic L, Zhou J, Jeong H, Then F (2005). Genome-wide expression profiling of human blood reveals biomarkers for Huntington's disease.. Proc Natl Acad Sci U S A.

[30] Maes OC, Xu S, Yu B, Chertkow HM, Wang E (2007). Transcriptional profiling of Alzheimer blood mononuclear cells by microarray.. Neurobiol Aging.

[31] Davenport RJ, Swingler RJ, Chancellor AM, Warlow CP (1996). Avoiding false positive diagnoses of motor neuron disease: lessons from the Scottish Motor Neuron Disease Register.. J Neurol Neurosurg Psychiatry.

[32] Traynor BJ, Codd MB, Corr B, Forde C, Frost E (2000). Amyotrophic lateral sclerosis mimic syndromes: a population-based study.. Arch Neurol.

[33] Kraemer M, Buerger M, Berlit P (2010). Diagnostic problems and delay of diagnosis in amyotrophic lateral sclerosis.. Clin Neurol Neurosurg.

[34] Srinivasan J, Scala S, Jones HR, Saleh F, Russell JA (2006). Inappropriate surgeries resulting from misdiagnosis of early amyotrophic lateral sclerosis.. Muscle Nerve.

[35] Staschke KA, Dong S, Saha J, Zhao J, Brooks NA (2009). IRAK4 kinase activity is required for Th17 differentiation and Th17-mediated disease.. J Immunol.

[36] Sharp FR, Xu H, Lit L, Walker W, Apperson M (2006). The future of genomic profiling of neurological diseases using blood.. Arch Neurol.

[37] Tang Y, Lu A, Aronow BJ, Sharp FR (2001). Blood genomic responses differ after stroke, seizures, hypoglycemia, and hypoxia: blood genomic fingerprints of disease.. Ann Neurol.

[38] Gladkevich A, Kauffman HF, Korf J (2004). Lymphocytes as a neural probe: potential for studying psychiatric disorders.. Prog Neuropsychopharmacol Biol Psychiatry.

[39] Basso M, Samengo G, Nardo G, Massignan T, D'Alessandro G (2009). Characterization of detergent-insoluble proteins in ALS indicates a causal link between nitrative stress and aggregation in pathogenesis.. PLoS ONE.

[40] Kim I, Xu W, Reed JC (2008). Cell death and endoplasmic reticulum stress: disease relevance and therapeutic opportunities.. Nat Rev Drug Discov.

[41] Beckman JS (1996). Oxidative damage and tyrosine nitration from peroxynitrite.. Chem Res Toxicol.

[42] Board PG, Coggan M, Chelvanayagam G, Easteal S, Jermiin LS (2000). Identification, characterization, and crystal structure of the Omega class glutathione transferases.. J Biol Chem.

[43] Ghezzi P, Casagrande S, Massignan T, Basso M, Bellacchio E (2006). Redox regulation of cyclophilin A by glutathionylation.. Proteomics.

[44] Milton RH, Abeti R, Averaimo S, DeBiasi S, Vitellaro L (2008). CLIC1 function is required for beta-amyloid-induced generation of reactive oxygen species by microglia.. J Neurosci.

[45] Wood ZA, Schroder E, Robin Harris J, Poole LB (2003). Structure, mechanism and regulation of peroxiredoxins.. Trends Biochem Sci.

[46] Beal MF, Ferrante RJ, Browne SE, Matthews RT, Kowall NW (1997). Increased 3-nitrotyrosine in both sporadic and familial amyotrophic lateral sclerosis.. Ann Neurol.

[47] Ferrante RJ, Shinobu LA, Schulz JB, Matthews RT, Thomas CE (1997). Increased 3-nitrotyrosine and oxidative damage in mice with a human copper/zinc superoxide dismutase mutation.. Ann Neurol.

[48] Barber SC, Mead RJ, Shaw PJ (2006). Oxidative stress in ALS: a mechanism of neurodegeneration and a therapeutic target.. Biochim Biophys Acta.

[49] Ilieva EV, Ayala V, Jove M, Dalfo E, Cacabelos D (2007). Oxidative and endoplasmic reticulum stress interplay in sporadic amyotrophic lateral sclerosis.. Brain.

[50] Krecic AM, Swanson MS (1999). hnRNP complexes: composition, structure, and function.. Curr Opin Cell Biol.

[51] Buratti E, Brindisi A, Giombi M, Tisminetzky S, Ayala YM (2005). TDP-43 binds heterogeneous nuclear ribonucleoprotein A/B through its C-terminal tail: an important region for the inhibition of cystic fibrosis transmembrane conductance regulator exon 9 splicing.. J Biol Chem.

[52] Lemmens R, Moore MJ, Al-Chalabi A, Brown RH, Robberecht W (2010). RNA metabolism and the pathogenesis of motor neuron diseases.. Trends Neurosci.

[53] Appel SH, Beers DR, Henkel JS (2010). T cell-microglial dialogue in Parkinson's disease and amyotrophic lateral sclerosis: are we listening?. Trends Immunol.

[54] Cedarbaum JM, Stambler N, Malta E, Fuller C, Hilt D (1999). The ALSFRS-R: a revised ALS functional rating scale that incorporates assessments of respiratory function. BDNF ALS Study Group (Phase III).. J Neurol Sci.

[55] Pappin DJ, Hojrup P, Bleasby AJ (1993). Rapid identification of proteins by peptide-mass fingerprinting.. Curr Biol.

[56] Carpi D, Korkalainen M, Airoldi L, Fanelli R, Hakansson H (2009). Dioxin-sensitive proteins in differentiating osteoblasts: effects on bone formation in vitro.. Toxicol Sci.

