# Supplementary figures and images for: Amyotrophic Lateral Sclerosis Multiprotein Biomarkers in Peripheral Blood Mononuclear Cells
Source: PLoS One. 2011 Oct 5;6(10):e25545. doi: 10.1371/journal.pone.0025545 (PMC3187793; doi:10.1371/journal.pone.0025545)

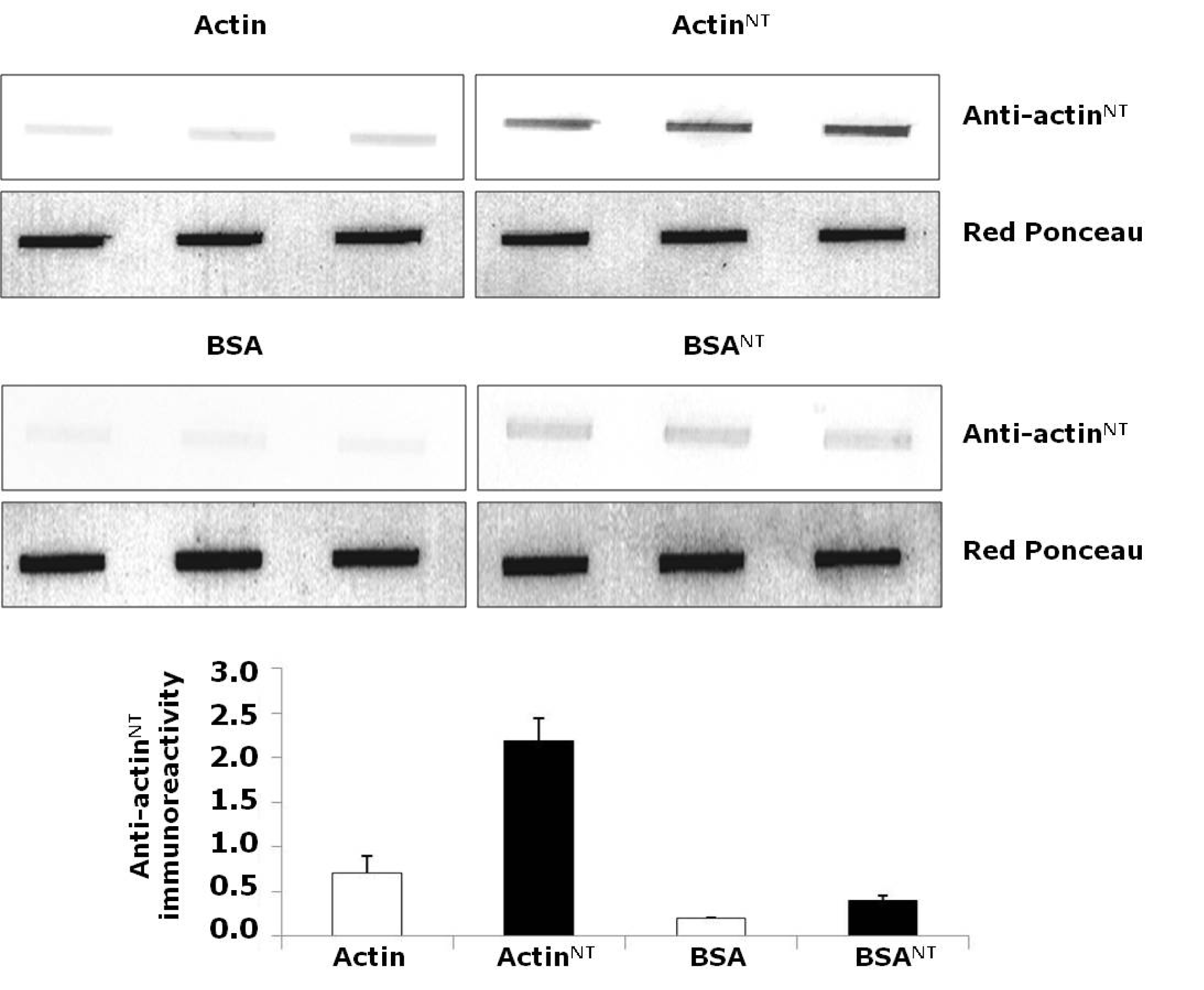

Supplement: Figure S1 — Anti-nitrated actin (actinNT) antibody preparation and characterization by dot blot assay. Purified human actin was nitrated in vitro and used as antigen in rabbits for raising polyclonal antibodies. The rabbit polyclonal antiserum was tested by dot blot with actin, actinNT, bovine serum albumin (BSA) and BSANT, prepared by the same procedure as actinNT. Figure S1 shows that anti-actinNT does not efficiently recognize another nitrated protein and has more than three times affinity for actinNT of unmodified actin. 3 µg of actin, actinNT, BSA and BSANT were loaded in each slot on the nitrocellulose membrane. The membrane was probed overnight with the polyclonal antiserum diluted 1∶7500. Immunoreactivity was normalized to the actual amount of protein loaded on the membrane as detected after Red Ponceau staining. (TIF) [file pone.0025545.s001.tif]

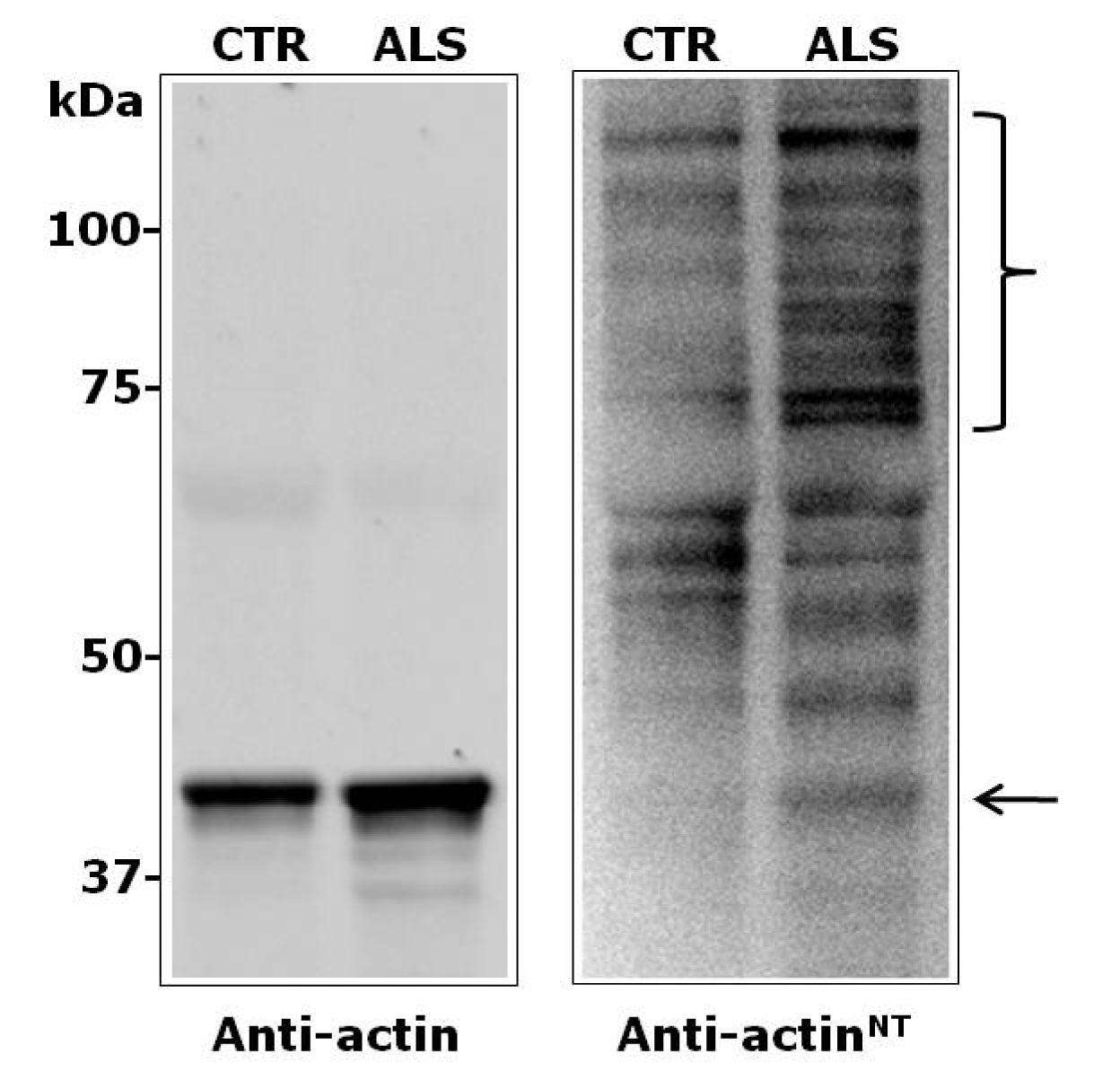

Supplement: Figure S2 — Characterization of the anti-actinNT antibody. The anti-actinNT antibody was further characterized to determine the specificity of the immunoreactivity against PBMC lysates from controls and ALS patients by Western blotting. Figure S2 shows that the antiserum recognized a band at 42 kDa, which is the expected Mw for actinNT, but also other bands at higher Mw. These are probably polymerized actin forms. These high-Mw species are essentially only detected in the patients and make this antibody especially useful to distinguish ALS patients from controls, also in a dot blot assay, as shown in Figure 1. Equal amounts of PBMC lysates (30 µg) from healthy controls and ALS patients were analyzed. A representative experiment is shown. The PVDF membrane was probed first with the anti-actin antibody (mouse monoclonal, 1∶1000 dilution, Chemicon), and the signal was revealed with a goat anti-mouse Qdot® 800-conjugated secondary antibody (Invitrogen, 1∶1000 dilution) on a laser scanner Molecular Imager FX (Bio-Rad), then with the anti-actinNT antibody, and the signal was revealed with an anti-rabbit peroxidase-conjugated secondary antibody/chemiluminescent HRP Substrate (Millipore) on a ChemiDoc XRS system (Bio-Rad). (TIF) [file pone.0025545.s002.tif]

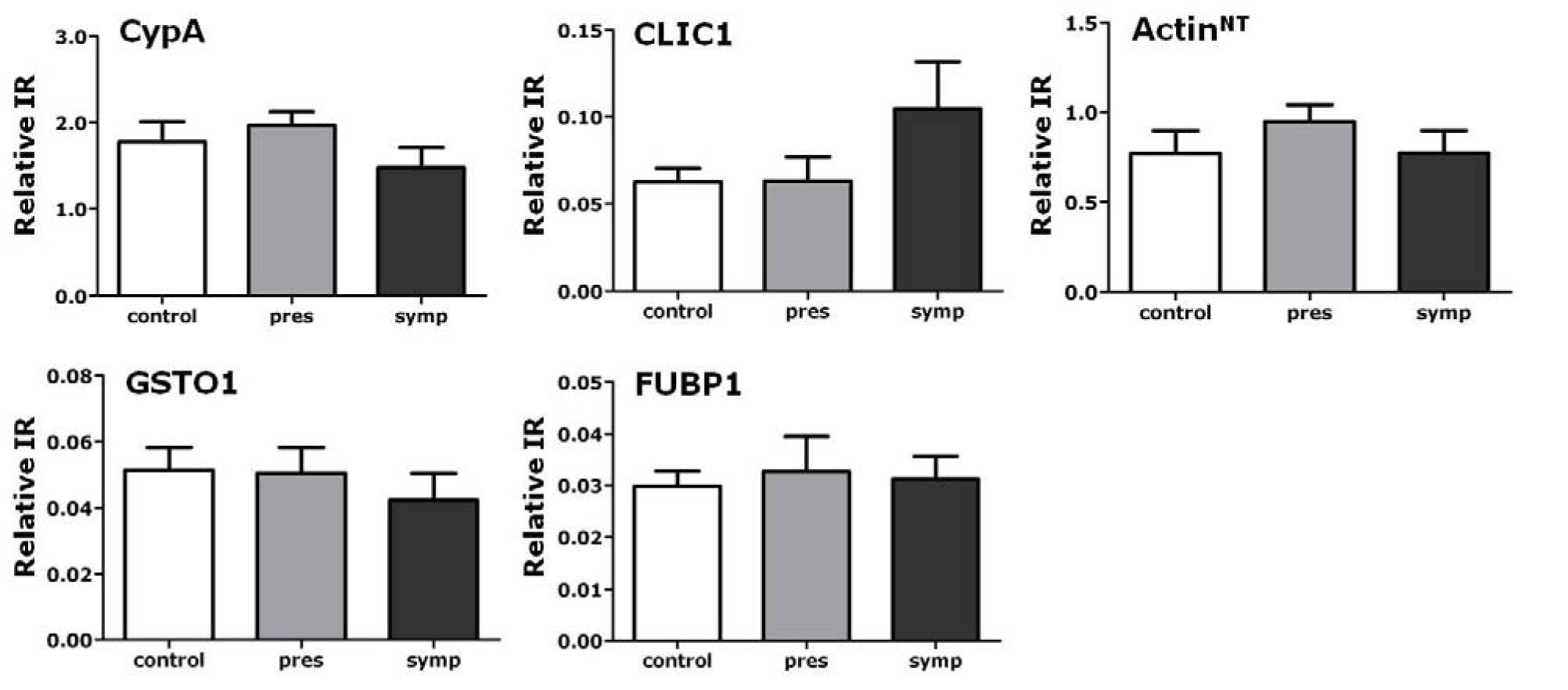

Supplement: Figure S3 — Analysis of protein biomarkers in dorsal horn spinal cord of the G93A SOD1 transgenic rat. Bars show relative immunoreactivity for the indicated proteins measured by dot blot in ventral horn tissue lysates of presymptomatic (pres, grey bars) (n = 6) and (symp, dark grey bars) G93A SOD1 (n = 6) rats and non transgenic (control, white bars) (n = 8) rats. Immunoreactivity was normalized to the actual amount of protein loaded, detected after Red Ponceau staining. None of the protein levels were significantly different from control (one-way ANOVA followed by Newman-Keuls multiple comparison test). (TIF) [file pone.0025545.s003.tif]
