# Supplementary material for: Amyotrophic Lateral Sclerosis Multiprotein Biomarkers in Peripheral Blood Mononuclear Cells
Source: PLoS One. 2011 Oct 5;6(10):e25545. doi: 10.1371/journal.pone.0025545 (PMC3187793; doi:10.1371/journal.pone.0025545)
Supplement: Table S1 — Main characteristics of healthy individuals and sALS patients used in the proteomic 2D DIGE analysis. (DOC) [file pone.0025545.s004.doc]

Table S1. Main characteristics of healthy individuals and sALS patients used in the proteomic 2D DIGE analysis.

| Sample | Clinical diagnosis | Age^1^ | Sex | Score^2^ | Onset^3^ | Duration^4^ | Survival^5^ |
| --- | --- | --- | --- | --- | --- | --- | --- |
| 1-11 | Healthy | 54±5 | 6(M), 5(F) | - | - | - | - |
| 12-22 | ALS | 65±13 | 5(M), 6(F) | >24 |  |  |  |
| 12 | ALS | 56 | F | 28/48 | spinal | 60 | >98* |
| 13 | ALS | 76 | M | 37/48 | spinal | 96 | 111 |
| 14 | ALS | 80 | F | 25/48 | n.a | 60 | 83 |
| 15 | ALS | 61 | M | 25/48 | bulbar | 36 | >72* |
| 16 | ALS | 39 | F | 39/48 | spinal | n.a. | n.a. |
| 17 | ALS | 66 | F | 27/48 | bulbar | 32 | >56* |
| 18 | ALS | 79 | M | 28/48 | spinal | 24 | 46 |
| 19 | ALS | 81 | F | 40/48 | bulbar | 24 | 51 |
| 20 | ALS | 59 | M | 33/48 | spinal | 9 | 30 |
| 21 | ALS | 52 | M | 45/48 | bulbar | 6 | 29 |
| 22 | ALS | 61 | F | 40/48 | spinal | 7 | >43* |
| 23-33 | ALS | 63±9 | 3(M), 8(F) | ≤24 |  |  |  |
| 23 | ALS | 73 | F | 15/48 | spinal | 17 | 24 |
| 24 | ALS | 52 | F | 24/48 | spinal | 60 | 75 |
| 25 | ALS | 64 | M | 24/48 | spinal | 43 | 70 |
| 26 | ALS | 63 | F | 22/48 | n.a. | 17 | 27 |
| 27 | ALS | 52 | F | 17/48 | bulbar | 8 | 11 |
| 28 | ALS | 72 | F | 12/48 | spinal | 27 | 29 |
| 29 | ALS | 61 | M | 12/48 | spinal | 28 | 30 |
| 30 | ALS | 72 | F | 21/48 | spinal | n.a. | n.a. |
| 31 | ALS | 47 | M | 17/48 | spinal | n.a. | n.a. |
| 32 | ALS | 63 | F | 23/48 | spinal | 16 | 31 |
| 33 | ALS | 72 | F | 24/48 | spinal | 24 | 26 |

^1^Age at PBMC collection; ^2^ALSFRS-R score at PBMC collection; ^3^Site of onset; ^4^Disease duration (months) from the onset of symptoms to PBMC collection; ^5^Disease duration (months) from the onset of symptoms to death; -, not applicable; *, patient still alive (Feb-2010); n.a, not available.
