# Supplementary material for: Amyotrophic Lateral Sclerosis Multiprotein Biomarkers in Peripheral Blood Mononuclear Cells
Source: PLoS One. 2011 Oct 5;6(10):e25545. doi: 10.1371/journal.pone.0025545 (PMC3187793; doi:10.1371/journal.pone.0025545)
Supplement: Table S2 — Spot volume changes in 2D DIGE analysis. (DOC) [file pone.0025545.s005.doc]

Table S2. Spot volume changes in 2D DIGE analysis.

| Spot volumes^1^ | ALS>24 vs ctr | ALS≤24 vs ctr | ALS>24 vs ALS≤24 |
| --- | --- | --- | --- |
| increased | 83 | 55 | 112 |
| decreased | 36 | 68 | 17 |
| unchanged | 107 | 103 | 97 |

^1^Normalized spot volumes. In the 2D DIGE analysis only the 226 matched spots in the three experimental groups were quantified. The spot volume was considered increased or decreased if the ratio of the volume in the patients to controls or in ALS>24 patients to ALS≤24 patients was ≥1.4; the spots with a ratio <1.4 were considered unchanged.
