# Supplementary material for: Amyotrophic Lateral Sclerosis Multiprotein Biomarkers in Peripheral Blood Mononuclear Cells
Source: PLoS One. 2011 Oct 5;6(10):e25545. doi: 10.1371/journal.pone.0025545 (PMC3187793; doi:10.1371/journal.pone.0025545)
Supplement: Table S3 — Candidate protein biomarkers identified by mass spectrometry. (DOC) [file pone.0025545.s006.doc]

Table S3. Candidate protein biomarkers identified by mass spectrometry.

| Spot | Uniprot ^1^ | Protein name | Mw_cal_ | pI_cal_ | Mw_obs_ | pI_obs_ | Sequence coverage | Matched peptides | Mascot score |
| --- | --- | --- | --- | --- | --- | --- | --- | --- | --- |
| 1 | P06576 | ATP synthase subunit beta (ATPase) | 56.5 | 5.2 | 60.0 | 4.9 | 46% | 21 | 156 |
| 2 | P60174 | Triosephosphate isomerase | 26.6 | 6.4 | 28.8 | 7.3 | 79% | 16 | 325 |
| 3 | P00558 | Phosphoglycerate kinase 1 | 44.5 | 8.3 | 54.4 | 8.5 | 72% | 36 | 506 |
| 4 | P00558 | Phosphoglycerate kinase 1* | 44.5 | 8.3 | 35.3 | 6.2 | 34% | 8 | 333 |
| 5 | P00558 | Phosphoglycerate kinase 1* | 44.5 | 8.3 | 35.1 | 5.8 | 16% | 4 | 147 |
| 6 | P18669 | Phosphoglycerate mutase 1* | 28.7 | 6.6 | 26.8 | 6.9 | 50% | 12 | 220 |
| 7 | Q8WU71 | Alpha-enolase | 47.1 | 7.0 | 55.4 | 6.3 | 31% | 12 | 173 |
| 8 | Q8WU71 | Alpha-enolase | 47.1 | 7.0 | 55.1 | 6.8 | 34% | 12 | 241 |
| 9 | Q8WU71 | Alpha-enolase | 47.1 | 7.0 | 55.1 | 7.0 | 43% | 20 | 113 |
| 10 | Q8WU71 | Alpha-enolase | 47.1 | 6.7 | 55.4 | 6.5 | 38% | 19 | 102 |
| 11-13 | P04075 | Fructose-bisphosphate aldolase A | 39.3 | 8.3 | 45.0 | 7.8/8.3/8.6 | 35% | 12 | 154 |
| 14 | P04406 | Glyceraldehyde-3-phosphate dehydrogenase | 35.9 | 8.5 | 36.9 | 8.4 | 53% | 16 | 161 |
| 15 | P00338 | L- Lactate dehydrogenase A chain# | 36.5 | 8.5 | 32.4 | 8.5 | 11% | 3 | 85 |
| 16 | P07195 | L-Lactate dehydrogenase B chain | 36.5 | 5.7 | 33.4 | 5.7 | 44% | 19 | 90 |
| 17 | P30043 | Flavin Reductase | 22.1 | 7.1 | 24.6 | 7.2 | 42% | 7 | 75 |
| 18 | P32119 | Peroxiredoxin-2 (PRDX2)# | 21.7 | 5.6 | 25.8 | 5.0 | 24% | 8 | 114 |
| 19 | P30041 | Peroxiredoxin-6 | 24.9 | 6.0 | 26.0 | 6.5 | 37% | 7 | 181 |
| 20 | P78417 | Glutathione S-transferase omega-1 (GSTO1) | 27.5 | 6.2 | 27.0 | 6.4 | 34 % | 13 | 95 |
| 21 | P04179 | Superoxide dismutase [Mn] | 24.7 | 8.3 | 24.7 | 7.4 | 34% | 6 | 62 |
| 22 | Q99497 | Protein DJ-1 | 19.8 | 6.3 | 23.4 | 6.1 | 42% | 5 | 70 |
| 23 | O00299 | Chloride intracellular channel protein 1 (CLIC1) | 28.7 | 5.7 | 28.0 | 4.9 | 63% | 14 | 130 |
| 24 | P11142 | Heat shock cognate 71 kDa protein (HSC70) | 71.0 | 5.3 | 70.0 | 5.1 | 36% | 25 | 161 |
| 25 | P11142 | HSC70* | 71.0 | 5.3 | 24.6 | 5.3 | 21% | 32 | 179 |
| 26 | P11021 | 78 kDa glucose-regulated protein | 72.2 | 5.0 | 75.0 | 4.9 | 11% | 10 | 87 |
| 27 | P62937 | Peptidyl-prolyl cis-trans isomerase A (CypA) | 18.0 | 7.7 | 18.7 | 7.8 | 61% | 11 | 84 |
| 28 | P07237 | Protein disulfide-isomerase (PDI) | 57.1 | 4.7 | 62.1 | 4.4 | 28% | 15 | 111 |
| 29 | P30101 | Protein disulfide-isomerase A3 (ERp57) | 56.7 | 6.0 | 60.6 | 5.4 | 13% | 9 | 125 |
| 30 | P30101 | ERP57 | 56.7 | 6.0 | 60.4 | 5.8 | 13% | 9 | 139 |
| 31 | P30101 | ERP57* | 56.7 | 6.0 | 50.0 | 6.2 | 32% | 25 | 121 |
| 32 | P30101 | ERP57* | 56.7 | 6.0 | 50.0 | 6.0 | 23% | 17 | 94 |
| 33 | P30040 | Endoplasmic reticulum protein ERp29 | 28.9 | 6.7 | 27.2 | 6.4 | 29% | 8 | 72 |
| 34 | P30040 | Endoplasmic reticulum protein ERp29 | 28.9 | 6.7 | 27.4 | 5.8 | 39% | 14 | 68 |
| 35 | P27797 | Calreticulin (CALR) | 48.1 | 4.2 | 55.8 | 3.9 | 27% | 11 | 90 |
| 36 | Q06323 | Proteasome activator complex subunit 1 (PA28a) | 28.7 | 5.7 | 29.1 | 5.4 | 46% | 11 | 84 |
| 37 | P60709 | Actin* | 42.0 | 5.3 | 38.0 | 5.1 | 42% | 19 | 149 |
| 38 | P60709 | Actin * | 42.0 | 5.3 | 38.0 | 5.3 | 46% | 19 | 380 |
| 39 | P60709 | Actin* | 42.0 | 5.3 | 26.6 | 4.9 | 22% | 7 | 257 |
| 40 | P60709 | Actin* | 42.0 | 5.3 | 26.5 | 5.3 | 18% | 6 | 270 |
| 41 | P60709 | Actin* | 42.0 | 5.3 | 27.8 | 5.2 | 30% | 13 | 317 |
| 42 | P60709 | Actin * | 42.0 | 5.3 | 26.5 | 5.1 | 23% | 8 | 210 |
| 43 | P60709 | Actin* | 42.0 | 5.3 | 26.5 | 4.8 | 62% | 26 | 378 |
| 44 | P18206 | Vinculin | 123.7 | 5.5 | 120 | 5.2 | 20% | 19 | 122 |
| 45 | P18206 | Vinculin* | 123.7 | 5.5 | 62.3 | 6.2 | 16% | 16 | 100 |
| 46 | P18206 | Vinculin | 123.7 | 5.5 | 126 | 5.7 | 57% | 74 | 468 |
| 47 | P18206 | Vinculin | 123.7 | 5.5 | 127 | 6.4 | 21% | 27 | 164 |
| 48 | P18206 | Vinculin | 123.7 | 5.5 | 130 | 6.2 | 12% | 15 | 100 |
| 49 | P26038 | Moesin | 67.7 | 6.0 | 71.0 | 6.3 | 25% | 14 | 83 |
| 50 | P26038 | Moesin | 67.7 | 6.0 | 71.0 | 6.7 | 36% | 23 | 237 |
| 51 | P26038 | Moesin | 67.7 | 6.0 | 71.0 | 7.0 | 7% | 4 | 71 |
| 52 | P67936 | Tropomyosin alpha-4 chain | 28.5 | 4.6 | 28.2 | 4.3 | 43% | 18 | 135 |
| 53 | P12814 | Alpha-Actinin-1 | 102.9 | 5.2 | 125 | 5.0 | 26% | 19 | 95 |
| 54 | P40121 | Actin-regulatory protein CAP-G | 38.4 | 5.8 | 40.0 | 5.9 | 16% | 5 | 67 |
| 55 | P40121 | Actin-regulatory protein CAP-G | 38.4 | 5.8 | 40.0 | 6.2 | 32% | 8 | 66 |
| 56 | P52907 | F-actin capping protein subunit-alpha 1 | 32.9 | 5.4 | 35.2 | 5.1 | 31% | 7 | 76 |
| 57 | Q9Y490 | Talin-1* | 269.7 | 5.7 | 121 | 5.2 | 2% | 5 | 72 |
| 58 | Q9Y490 | Talin-1* | 269.7 | 5.7 | 38.0 | 6.6 | 2% | 13 | 83 |
| 59 | P37802 | Transgelin-2 | 22.3 | 8.4 | 21.7 | 8.3 | 41% | 9 | 82 |
| 60 | P21333 | Filamin-A*# | 280.6 | 5.7 | 110 | 5.7 | 2% | 7 | 124 |
| 61 | Q14789 | Giantin* | 375.7 | 4.9 | 110 | 5.0 | 20% | 83 | 64 |
| 62 | Q9BZM1 | Group XIIA secretory phospholipase A2 | 21.0 | 6.9 | 26.0 | 6.8 | 26% | 5 | 65 |
| 63 | P07355 | Annexin A2# | 38.5 | 7.5 | 38.8 | 7.8 | 26% | 10 | 228 |
| 64 | P30740 | Leukocyte elastase inhibitor | 42.7 | 5.9 | 36.5 | 6.3 | 17% | 5 | 64 |
| 65 | Q9NWZ3 | Interleukin-1 receptor-associated kinase 4 (IRAK4)* | 51.5 | 5.2 | 25.1 | 6.2 | 31% | 16 | 59 |
| 66 | Q96AE4 | Far upstream element-binding protein 1 (FUBP1) | 67.5 | 7.1 | 71.0 | 6.9 | 29% | 19 | 166 |
| 67 | Q96AE4 | FBP1 | 67.5 | 7.1 | 71.0 | 7.2 | 20% | 12 | 74 |
| 68 | P22627 | Heterogeneous nuclear ribonucleoproteins A2/B1(ROA2) | 37.4 | 8.9 | 38.4 | 8.9 | 29% | 7 | 404 |
| 69 | Q9UJV9 | Probable ATP-dependent RNA helicase DDX41 | 69.7 | 6.4 | 56.0 | 6.8 | 22% | 14 | 72 |
| 70 | O00170 | AH receptor-interacting protein | 37.6 | 6.0 | 40.0 | 7.0 | 27% | 10 | 60 |
| 71 | Q96BD8 | Spindle and kinetochore-associated protein 1 | 28.9 | 6.7 | 27.7 | 5.6 | 25% | 6 | 64 |

^1^Entry from the UniProt Knowledgebase database; Mw_calc_ and pI_calc_, calculated Mw and pI; Mw_obs_ and pI_obs_, observed Mw and pI; *, protein with lower than expected Mw, possibly a fragment. Proteins were identified based on combined MS and MS/MS on a 4800 MALDI TOF/TOF mass spectrometer (Applied Biosystems) except those indicated by #. These were identified by LC-MS/MS using the microfluid chip-based technology for nanoelectrospray coupled to an ion trap mass spectrometer (Agilent 1200 LC/MSD Trap XCT); MASCOT score, MASCOT protein score derived from the combination of MS and MS/MS data.
