# Supplementary material for: Amyotrophic Lateral Sclerosis Multiprotein Biomarkers in Peripheral Blood Mononuclear Cells
Source: PLoS One. 2011 Oct 5;6(10):e25545. doi: 10.1371/journal.pone.0025545 (PMC3187793; doi:10.1371/journal.pone.0025545)
Supplement: Table S4 — Candidate protein biomarkers: 2D DIGE quantitative analysis. (DOC) [file pone.0025545.s007.doc]

Table S4. Candidate protein biomarkers: 2D DIGE quantitative analysis.

| Spot | Protein name | ALS>24 vs controls (fc)^1^ | ALS≤24 vs controls (fc) |
| --- | --- | --- | --- |
| Energy metabolism | | | |
| 1 | ATP synthase subunit beta | 1.6 | -1.7 |
| 2 | Triosephosphate isomerase | 1.7 | n.c. |
| 3 | Phosphoglycerate kinase 1 | 1.6 | -2.0 |
| 4 | Phosphoglycerate kinase 1* | 3.3 | 4.3 |
| 5 | Phosphoglycerate kinase 1* | 4.5 | 3.0 |
| 6 | Phosphoglycerate mutase 1* | 1.7 | 1.5 |
| 7 | Alpha-enolase | 3.1 | n.c. |
| 8 | Alpha-enolase | 2.5 | n.c. |
| 9 | Alpha-enolase | 2.0 | n.c. |
| 10 | Alpha-enolase | 2.7 | n.c. |
| 11-13 | Fructose-bisphosphate aldolase A | 3.2 | 1.5 |
| 14 | Glyceraldehyde-3-phosphate dehydrogenase | 1.9 | n.c. |
| 15 | L- Lactate dehydrogenase A chain | n.c. | -1.4 |
| 16 | L-Lactate dehydrogenase B chain | 2.8 | n.c. |
| Redox regulation | | | |
| 17 | Flavin Reductase | -1.7 | n.c. |
| 18 | PRDX2 | 1.7 | 2.8 |
| 19 | Peroxiredoxin-6 | 2.7 | 1.4 |
| 20 | GSTO1 | n.c. | -1.5 |
| 21 | Superoxide dismutase [Mn] | n.c. | 1.4 |
| 22 | Protein DJ-1 | 2.4 | 3.6 |
| 23 | CLIC1 | 1.5 | 2.0 |
| Protein folding and degradation | | | |
| 24 | HSC70 | 1.5 | n.c. |
| 25 | HSC70* | 2.8 | 2.5 |
| 26 | 78 kDa glucose-regulated protein | nc | -2.9 |
| 27 | CypA | 1.5 | n.c. |
| 28 | PDI | 1.4 | 1.6 |
| 29 | ERp57 | -1.4 | -1.8 |
| 30 | ERp57 | n.c. | 2.0 |
| 31 | ERp57* | 2.0 | 2.0 |
| 32 | ERp57* | 2.0 | n.c. |
| 33 | Endoplasmic reticulum protein ERp29 | 1.8 | 1.8 |
| 34 | Endoplasmic reticulum protein ERp29 | 1.6 | 1.6 |
| 35 | CALR | 2.5 | n.c. |
| 36 | PA28a | -1.4 | -1.7 |
| Cytoskeleton-associated | | | |
| 37 | Actin* | n.c. | 2.0 |
| 38 | Actin* | 3.6 | n.c. |
| 39 | Actin* | 2.3 | 3.2 |
| 40 | Actin* | 1.5 | 2.8 |
| 41 | Actin* | 1.8 | 3.5 |
| 42 | Actin * | 1.7 | 2.5 |
| 43 | Actin* | 2.5 | 3.9 |
| 44 | Vinculin* | n.c. | -1.5 |
| 45 | Vinculin* | 1.5 | -1.5 |
| 46 | Vinculin | 3.4 | 2.3 |
| 47 | Vinculin | 3.4 | 2.3 |
| 48 | Vinculin | -2.4 | -2.8 |
| 49 | Moesin | -2.2 | -1.5 |
| 50 | Moesin | -1.5 | -1.6 |
| 51 | Moesin | -1.7 | -1.9 |
| 52 | Tropomyosin alpha-4 chain | 3.0 | n.c. |
| 53 | Alpha-Actinin-1 | -1.9 | -2.0 |
| 54 | Actin-regulatory protein CAP-G | 1.4 | n.c. |
| 55 | Actin-regulatory protein CAP-G | 2.8 | 2.9 |
| 56 | F-acti capping protein subunit-alpha 1 | 1.9 | 1.4 |
| 57 | Talin-1* | 2.5 | 2.7 |
| 58 | Talin-1* | 3.4 | 3.0 |
| 59 | Transgelin-2 | 2.7 | 3.5 |
| 60 | Filamin-A | -1.5 | -1.5 |
| 61 | Giantin | -1.8 | -1.8 |
| Inflammatory response | | | |
| 62 | Group XIIA secretory phospholipase A2 | 1.5 | 1.8 |
| 63 | Annexin A2 | 2.7 | 1.4 |
| 64 | Leukocyte elastase inhibitor | 4.6 | 2.2 |
| 65 | IRAK4* | 2.7 | n.c. |
| DNA/RNA binding | | | |
| 66 | FUBP1 | -2.0 | -3.5 |
| 67 | FUBP1 | n.c. | -1.8 |
| 68 | ROA2 | 1.6 | n.c. |
| 69 | Probable ATP-dependent RNA helicase DDX41 | 2.7 | 2.9 |
| Others | | | |
| 70 | AH receptor-interacting protein | 2.6 | 3.2 |
| 71 | Spindle and kinetochore-associated protein 1 | 2.0 | 3.5 |

^1^fc, -fold change: the mean of two 2D DIGE experiments (dye-swap normalization) for increase (positive) and decrease (negative) of normalized spot volumes in samples from ALS patients, ALS>24 (pool of 11) and ALS≤24 (pool of 11), in comparison with healthy controls (pool of 11); n.c., no changed, -fold changes <1.4; only -fold changes ≥1.4 are reported. *, protein with lower than expected Mw, probably a fragment.
