# Supplementary material for: Amyotrophic Lateral Sclerosis Multiprotein Biomarkers in Peripheral Blood Mononuclear Cells
Source: PLoS One. 2011 Oct 5;6(10):e25545. doi: 10.1371/journal.pone.0025545 (PMC3187793; doi:10.1371/journal.pone.0025545)
Supplement: Table S5 — Main characteristics of healthy individuals and sALS patients used for the validation analysis ( Figure 2A and Figure 3 ). (DOC) [file pone.0025545.s008.doc]

Table S5. Main characteristics of healthy individuals and sALS patients used for the validation analysis (Figure 2A and Figure 3).

| Sample | Clinical diagnosis | Age^1^ | Sex | Score^2^ | Onset^3^ | Duration^4^ | Survival^5^ |
| --- | --- | --- | --- | --- | --- | --- | --- |
| 1-30 | Healthy | 46±9 | 18(M), 12(F) | - | - | - | - |
| 31-60 | ALS | 62±11 | 15(M), 15(F) | >24 |  |  |  |
| 31 | ALS | 50 | F | 32/48 | spinal | n.a. | n.a. |
| 32 | ALS | 61 | M | 32/48 | spinal | 29 | 41 |
| 33 | ALS | 76 | M | 37/48 | spinal | 96 | 111 |
| 34 | ALS | 56 | F | 28/48 | spinal | 60 | >98* |
| 35 | ALS | 74 | F | 28/48 | bulbar | 17 | 52 |
| 36 | ALS | 49 | M | 38/48 | spinal | 5 | 32 |
| 37 | ALS | 63 | F | 32/48 | bulbar | 16 | 30 |
| 38 | ALS | 52 | M | 34/48 | bulbar | 28 | 33 |
| 39 | ALS | 66 | F | 27/48 | bulbar | 32 | >56* |
| 40 | ALS | 39 | F | 39/48 | spinal | n.a. | n.a. |
| 41 | ALS | 59 | M | 33/48 | spinal | 9 | 30 |
| 42 | ALS | 58 | M | 43/48 | bulbar | 33 | 59 |
| 43 | ALS | 64 | M | 29/48 | spinal | 38 | 43 |
| 44 | ALS | 68 | F | 40/48 | spinal | n.a. | n.a. |
| 45 | ALS | 40 | F | 40/48 | spinal | 20 | >43* |
| 46 | ALS | 59 | M | 28/48 | spinal | 25 | >43* |
| 47 | ALS | 55 | M | 33/48 | spinal | 28 | 57 |
| 48 | ALS | 72 | M | 35/48 | spinal | 13 | >34* |
| 49 | ALS | 69 | F | 26/48 | bulbar | 26 | 40 |
| 50 | ALS | 53 | F | 28/48 | spinal | 19 | 21 |
| 51 | ALS | 62 | M | 29/48 | spinal | 108 | >131* |
| 52 | ALS | 50 | F | 27/48 | spinal | 7 | 9 |
| 53 | ALS | 83 | F | 25/48 | spinal | 35 | >62* |
| 54 | ALS | 72 | M | 25/48 | spinal | n.a. | n.a. |
| 55 | ALS | 52 | M | 45/48 | bulbar | 6 | 29 |
| 56 | ALS | 79 | M | 28/48 | spinal | 24 | 46 |
| 57 | ALS | 81 | F | 40/48 | bulbar | 24 | 51 |
| 58 | ALS | 61 | F | 40/48 | spinal | 7 | >43* |
| 59 | ALS | 77 | F | 27/48 | bulbar | 32 | >56* |
| 60 | ALS | 61 | M | 25/48 | bulbar | 36 | >72* |
| 61-90 | ALS | 62±9 | 12(M), 18(F) | ≤24 |  |  |  |
| 61 | ALS | 61 | M | 12/48 | spinal | 28 | 30 |
| 62 | ALS | 60 | M | 21/48 | bulbar | 17 | 25 |
| 63 | ALS | 69 | F | 24/48 | bulbar | 22 | 34 |
| 64 | ALS | 63 | M | 17/48 | spinal | 24 | 40 |
| 65 | ALS | 62 | M | 19/48 | spinal | 6 | 11 |
| 66 | ALS | 65 | M | 22/48 | spinal | 30 | 66 |
| 67 | ALS | 64 | M | 24/48 | spinal | 43 | 70 |
| 68 | ALS | 72 | F | 12/48 | spinal | 27 | 29 |
| 69 | ALS | 55 | F | 13/48 | spinal | 51 | 61 |
| 70 | ALS | 48 | F | 16/48 | spinal | 19 | >55* |
| 71 | ALS | 42 | F | 17/48 | spinal | 38 | 66 |
| 72 | ALS | 49 | F | 24/48 | spinal | 24 | 54 |
| 73 | ALS | 73 | F | 15/48 | spinal | 17 | 24 |
| 74 | ALS | 56 | F | 10/48 | bulbar | 48 | 57 |
| 75 | ALS | 59 | F | 10/48 | spinal | 32 | 54 |
| 76 | ALS | 73 | F | 23/48 | spinal | 21 | >43* |
| 77 | ALS | 48 | F | 19/48 | spinal | 10 | 29 |
| 78 | ALS | 70 | F | 24/48 | bulbar | 60 | >55* |
| 79 | ALS | 63 | M | 11/48 | spinal | 42 | 86 |
| 80 | ALS | 64 | F | 21/48 | spinal | 22 | 30 |
| 81 | ALS | 52 | F | 24/48 | spinal | 60 | 75 |
| 82 | ALS | 74 | M | 17/48 | spinal | n.a. | n.a. |
| 83 | ALS | 63 | F | 23/48 | spinal | 16 | 31 |
| 84 | ALS | 69 | F | 22/48 | bulbar | 19 | 49 |
| 85 | ALS | 63 | M | 10/48 | spinal | 10 | 13 |
| 86 | ALS | 72 | F | 21/48 | spinal | n.a. | n.a. |
| 87 | ALS | 71 | F | 20/48 | bulbar | 16 | 20 |
| 88 | ALS | 71 | M | 24/48 | spinal | 24 | 26 |
| 89 | ALS | 52 | F | 17/48 | bulbar | 8 | 11 |
| 90 | ALS | 47 | M | 17/48 | spinal | n.a. | n.a. |

^1^Age at PBMC collection; ^2^ALSFRS-R score at PBMC collection; ^3^Site of onset; ^4^Disease duration (months) from the onset of symptoms to PBMC collection; ^5^Disease duration (months) from the onset of symptoms to death; -, not applicable; *, patient still alive (Feb-2010); n.a, not available.
