# Supplementary material for: Amyotrophic Lateral Sclerosis Multiprotein Biomarkers in Peripheral Blood Mononuclear Cells
Source: PLoS One. 2011 Oct 5;6(10):e25545. doi: 10.1371/journal.pone.0025545 (PMC3187793; doi:10.1371/journal.pone.0025545)
Supplement: Table S6 — Univariate logistic regression: controls versus ALS>24. (DOC) [file pone.0025545.s009.doc]

| Table S6. Univariate logistic regression: controls versus ALS>24. | | | | | |
| --- | --- | --- | --- | --- | --- |
| Protein | OR | 95% CI | | P-value | AUC |
| CALR | 2.002 | 1.268 | 3.163 | 0.0029 | 0.711 |
| TDP-43 | 1.377 | 1.010 | 1.877 | 0.0430 | 0.657 |
| PRDX2 | 6.600 | 1.807 | 24.101 | 0.0043 | 0.723 |
| PDI | 48.689 | 4.370 | 542.533 | 0.0016 | 0.818 |
| ERp57 | 4.819 | 1.940 | 11.971 | 0.0007 | 0.816 |
| PA28a | 0.608 | 0.412 | 0.898 | 0.0123 | 0.721 |
| CLIC1 | 39.908 | 5.412 | 294.281 | 0.0003 | 0.794 |
| IRAK4 | 13.379 | 3.207 | 55.810 | 0.0004 | 0.852 |
| FUBP1 | 0.283 | 0.115 | 0.695 | 0.0059 | 0.806 |
| GSTO1 | 0.278 | 0.098 | 0.785 | 0.0157 | 0.701 |
| HSC70 | 4.167 | 1.664 | 10.437 | 0.0023 | 0.742 |
| CypA | 3.312 | 1.516 | 7.235 | 0.0027 | 0.787 |
| Actin^NT^ | 79.972 | 7.635 | 837.669 | 0.0003 | 0.864 |
| ROA2 | 1.865 | 1.305 | 2.664 | 0.0006 | 0.792 |

Results are expressed as odds ratios (OR) and 95% confidence intervals (95% CI). A 95% CI not including the value of 1 indicates a statistically significant result. All probability values were two-sided and p<0.05 was considered statistically significant (values in bold type).
