# Supplementary material for: Amyotrophic Lateral Sclerosis Multiprotein Biomarkers in Peripheral Blood Mononuclear Cells
Source: PLoS One. 2011 Oct 5;6(10):e25545. doi: 10.1371/journal.pone.0025545 (PMC3187793; doi:10.1371/journal.pone.0025545)
Supplement: Table S7 — Univariate logistic regression: controls versus ALS≤24. (DOC) [file pone.0025545.s010.doc]

| Table S7. Univariate logistic regression: controls versus ALS≤24. | | | | | |
| --- | --- | --- | --- | --- | --- |
| Protein | OR | 95% CI | | P-value | AUC |
| CALR | 2.863 | 1.367 | 5.995 | 0.0053 | 0.752 |
| TDP-43 | 1.859 | 1.320 | 2.620 | 0.0004 | 0.810 |
| PRDX2 | 1.027 | 1.012 | 1.041 | 0.0003 | 0.836 |
| PDI | 1.043 | 1.018 | 1.068 | 0.0006 | 0.843 |
| ERp57 | 1.020 | 1.008 | 1.032 | 0.0007 | 0.893 |
| PA28a | 0.707 | 0.527 | 0.949 | 0.0209 | 0.715 |
| CLIC1 | 1.036 | 1.016 | 1.057 | 0.0004 | 0.794 |
| IRAK4 | 7.887 | 2.262 | 27.497 | 0.0012 | 0.835 |
| FUBP1 | 0.608 | 0.379 | 0.976 | 0.0392 | 0.723 |
| GSTO1 | 0.061 | 0.010 | 0.354 | 0.0019 | 0.771 |
| HSC70 | 6.794 | 2.342 | 19.709 | 0.0004 | 0.822 |
| CypA | 1.830 | 1.001 | 3.345 | 0.0497 | 0.620 |
| Actin^NT^ | 1.059 | 1.026 | 1.093 | 0.0003 | 0.937 |
| ROA2 | 1.931 | 1.359 | 2.745 | 0.0002 | 0.832 |

Results are expressed as odds ratios (OR) and 95% confidence intervals (95% CI). A 95% CI not including the value of 1 indicates a statistically significant result. All probability values were two-sided and p<0.05 was considered statistically significant (values in bold type).
