# Supplementary material for: Amyotrophic Lateral Sclerosis Multiprotein Biomarkers in Peripheral Blood Mononuclear Cells
Source: PLoS One. 2011 Oct 5;6(10):e25545. doi: 10.1371/journal.pone.0025545 (PMC3187793; doi:10.1371/journal.pone.0025545)
Supplement: Table S8 — Characteristics of sALS patients and non-ALS neurological controls used for the validation analysis ( Figure 2B ). (DOC) [file pone.0025545.s011.doc]

Table S8. Characteristics of sALS patients and non-ALS neurological controls used for the validation analysis (Figure 2B).

| Sample | Clinical diagnosis | Age^1^ | Sex | Score^2^ | Onset^3^ | Duration^4^ | Survival^5^ |
| --- | --- | --- | --- | --- | --- | --- | --- |
| 1-20 | ALS | 65±9 | 10(M), 10(F) | >24 |  |  |  |
| 1 | ALS | 76 | M | 32/48 | bulbar | 16 | 30 |
| 2 | ALS | 58 | M | 25/48 | spinal | 60 | 61 |
| 3 | ALS | 71 | F | 30/48 | spinal | 27 | 48 |
| 4 | ALS | 61 | M | 25/48 | bulbar | 36 | >72* |
| 5 | ALS | 63 | M | 26/48 | bulbar | 25 | 30 |
| 6 | ALS | 52 | M | 25/48 | spinal | 36 | 58 |
| 7 | ALS | 67 | M | 39/48 | spinal | 25 | 30 |
| 8 | ALS | 56 | F | 32/48 | bulbar | 36 | 51 |
| 9 | ALS | 53 | M | 25/48 | spinal | 16 | 31 |
| 10 | ALS | 77 | F | 27/48 | spinal | 7 | 9 |
| 11 | ALS | 75 | F | 35/48 | spinal | 24 | 44 |
| 12 | ALS | 77 | F | 26/48 | bulbar | 26 | 40 |
| 13 | ALS | 66 | F | 27/48 | bulbar | 32 | >56* |
| 14 | ALS | 68 | F | 40/48 | spinal | n.a. | n.a. |
| 15 | ALS | 68 | M | 28/48 | bulbar | 16 | 27 |
| 16 | ALS | 67 | F | 34/48 | spinal | 5 | 11 |
| 17 | ALS | 70 | F | 25/48 | spinal | 2 | 15 |
| 18 | ALS | 58 | M | 45/48 | spinal | 4 | 15 |
| 19 | ALS | 72 | M | 30/48 | bulbar | 9 | 17 |
| 20 | ALS | 66 | F | 35/48 | spinal | 10 | 24 |
| 21-43 | Neurological controls | 64±14 | 13(M), 10(F) | - | - | - | - |
| 21 | diabetic peripheral neuropathy | 79 | M | - | - | - | - |
| 22 | diabetic peripheral neuropathy | 80 | F | - | - | - | - |
| 23 | diabetic peripheral neuropathy | 70 | F | - | - | - | - |
| 24 | CIDP^6^ | 79 | F | - | - | - | - |
| 25 | CIDP | 61 | M | - | - | - | - |
| 26 | CIDP | 62 | M | - | - | - | - |
| 27 | CIDP | 81 | M | - | - | - | - |
| 28 | alcoholic polyneuropathy | 38 | M | - | - | - | - |
| 29 | alcoholic polyneuropathy | 49 | M | - | - | - | - |
| 30 | alcoholic polyneuropathy | 60 | M | - | - | - | - |
| 31 | axonal peripheral neuropathy | 79 | M | - | - | - | - |
| 32 | axonal peripheral neuropathy | 76 | F | - | - | - | - |
| 33 | axonal peripheral neuropathy | 69 | F | - | - | - | - |
| 34 | Charcot-Marie-Tooth disease | 38 | F | - | - | - | - |
| 35 | spinocerebellar ataxia | 43 | F | - | - | - | - |
| 36 | multiple sclerosis | 51 | F | - | - | - | - |
| 37 | multiple sclerosis | 52 | F | - | - | - | - |
| 38 | multiple sclerosis | 59 | F | - | - | - | - |
| 39 | myelopathy localized at C6-C7 | 61 | M | - | - | - | - |
| 40 | transverse myelitis | 63 | M | - | - | - | - |
| 41 | transverse myelitis | 73 | M | - | - | - | - |
| 42 | polymyositis | 74 | M | - | - | - | - |
| 43 | Lambert-Eaton myasthenic syndrome | 77 | M | - | - | - | - |

^1^Age at PBMC collection; ^2^ALSFRS-R score at PBMC collection; ^3^Site of onset; ^4^Disease duration (months) from the onset of symptoms to PBMC collection; ^5^Disease duration (months) from the onset of symptoms to death; ^6^CIDP, chronic inflammatory demyelinating polyneuropathy; -, not applicable; *, patient still alive (Feb-2010); n.a, not available.
