# Supplementary material for: Amyotrophic Lateral Sclerosis Multiprotein Biomarkers in Peripheral Blood Mononuclear Cells
Source: PLoS One. 2011 Oct 5;6(10):e25545. doi: 10.1371/journal.pone.0025545 (PMC3187793; doi:10.1371/journal.pone.0025545)
Supplement: Table S9 — Characteristics of sALS patients used for the longitudinal study ( Figure 4 ). (DOC) [file pone.0025545.s012.doc]

Table S9. Characteristics of sALS patients used for the longitudinal study (Figure 4).

| Sample | Clinical diagnosis | Age^1^ | Sex | Score T1^2^ | ScoreT2^2^ | Score T3^2^ | Site of Onset | Disease duration^3^ |
| --- | --- | --- | --- | --- | --- | --- | --- | --- |
| 1-13 | ALS |  |  |  |  |  |  |  |
| 1 | ALS | 38 | M | 41 | 34 | 30 | spinal | 5 |
| 2 | ALS | 64 | M | 30 | 20 | 19 | bulbar | 47 |
| 3 | ALS | 80 | M | 24 | 22 | 23 | bulbar | 19 |
| 4 | ALS | 73 | M | 42 | 37 | 36 | spinal | 15 |
| 5 | ALS | 69 | M | 37 | 31 | 30 | bulbar | 16 |
| 6 | ALS | 63 | M | 34 | 31 | 24 | bulbar | 21 |
| 7 | ALS | 69 | M | 35 | 27 | 27 | spinal | 10 |
| 8 | ALS | 64 | M | 28 | 23 | 12 | bulbar | 22 |
| 9 | ALS | 59 | F | 30 | 29 | 31 | spinal | 18 |
| 10 | ALS | 71 | F | 33 | 28 | 13 | spinal | 26 |
| 11 | ALS | 57 | F | 38 | 35 | 32 | spinal | 23 |
| 12 | ALS | 74 | M | 28 | 21 | 16 | bulbar | 35 |
| 13 | ALS | 60 | F | 31 | 29 | 30 | spinal | 20 |

^1^Age at first PBMC collection; ^2^ALSFRS-R score at PBMC collection: at t=0 (T1), at t=3 months (T2) and at t=6 months (T3); ^3^Disease duration (months) from the onset of symptoms to first PBMC collection.
